# Investigating Pleiotropy Between Depression and Autoimmune Diseases Using the UK Biobank

**DOI:** 10.1016/j.bpsgos.2021.03.002

**Published:** 2021-06

**Authors:** Kylie P. Glanville, Jonathan R.I. Coleman, Paul F. O'Reilly, James Galloway, Cathryn M. Lewis

**Affiliations:** a Social Genetic and Developmental Psychiatry Centre, Institute of Psychiatry, Psychology and Neuroscience, King's College London, London, United Kingdom; b NIHR Biomedical Research Centre, South London and Maudsley NHS Trust, King's College London, London, United Kingdom; c Department of Inflammation Biology, King's College London, London, United Kingdom; d Department of Medical and Molecular Genetics, King's College London, London, United Kingdom; e Department Genetics and Genomic Sciences, Icahn School of Medicine, Mount Sinai, New York, New York

**Keywords:** Autoimmune diseases, Depression, Genetics, Pleiotropy, Autoimmune diseases, Depression, Genetics, Pleiotropy, UK Biobank

## Abstract

**Background:**

Epidemiological studies report increased comorbidity between depression and autoimmune diseases. The role of shared genetic influences in the observed comorbidity is unclear. We investigated the evidence for pleiotropy between these traits in the UK Biobank (UKB).

**Methods:**

We defined autoimmune and depression cases using hospital episode statistics, self-reported conditions and medications, and mental health questionnaires. Pairwise comparisons of depression prevalence between autoimmune cases and controls, and vice versa, were performed. Cross-trait polygenic risk score (PRS) analyses tested for pleiotropy, i.e., whether PRSs for depression could predict autoimmune disease status, and vice versa.

**Results:**

We identified 28,479 cases of autoimmune diseases (pooling across 14 traits) and 324,074 autoimmune controls, and 65,075 cases of depression and 232,552 depression controls. The prevalence of depression was significantly higher in autoimmune cases than in controls, and similarly, the prevalence of autoimmune disease was higher in depression cases than in controls. PRSs for myasthenia gravis and psoriasis were significantly higher in depression cases than in controls (*p <* 5.2 × 10^−5^, *R*^2^ ≤ 0.04%). PRSs for depression were significantly higher in inflammatory bowel disease, psoriasis, psoriatic arthritis, rheumatoid arthritis, and type 1 diabetes cases than in controls (*p <* 5.8 × 10^−5^, *R*^2^ range = 0.06%–0.27%), and lower in celiac disease cases than in controls (*p <* 5.4 × 10^−7^, *R*^2^ range = 0.11%–0.15%).

**Conclusions:**

Consistent with the literature, depression was more common in individuals with autoimmune diseases than in controls, and vice versa. PRSs showed some evidence for involvement of shared genetic factors, but the modest *R*^2^ values suggest that shared genetic architecture accounts for a small proportion of the increased risk across traits.

There is evidence that individuals with a history of autoimmune disease are at greater risk for developing depression (1, 2, 3, 4) and that a history of depression increases risk for developing autoimmune diseases (5,6). The mechanisms driving the bidirectional relationship are poorly understood, but one contributory factor may be that these diseases share biological pathways. We and others have shown that there is no strong evidence for the involvement of human leukocyte antigen alleles in risk for depression, suggesting that the major histocompatibility complex does not harbor shared risk for depression and autoimmune diseases (6, 7, 8). However, genetic risk for autoimmune diseases occurs across the genome (9), and pleiotropic effects outside the major histocompatibility complex may be involved in shared risk for depression and autoimmune diseases.

Few studies have investigated evidence for genome-wide pleiotropy between depression and autoimmune diseases. Euesden *et al.* (10) found no evidence for association between polygenic risk scores (PRSs) for depression and risk for rheumatoid arthritis (RA), or vice versa. The Psychiatric Genomics Consortium indicated no evidence for significant genetic correlations (*r*_G_) between depression and nine autoimmune diseases (after multiple testing correction across 221 traits in total); the strongest correlation observed was between depression and inflammatory bowel disease (IBD) (*r*_G_ = .07, uncorrected *p =* .01) (11). Recently, Liu *et al.* (6) found no association between PRSs for mental health disorders and risk for autoimmune diseases, and only a weak association between PRSs for autoimmune diseases and risk for mental health disorders.

We extend previous work, leveraging the UK Biobank (UKB) to test for pleiotropy between depression and autoimmune diseases with PRS methodology. Given the challenge of reliably defining complex traits using large-scale data, we took two approaches to defining autoimmune diseases and depression. We classified liberally defined cases, based on a single item endorsing diagnosis with an autoimmune disease, and strictly defined cases, based on multiple items. We used this approach to identify individuals affected by any of 14 autoimmune or autoinflammatory traits—collectively referred to as autoimmune diseases throughout. We took a similar approach to classifying depression by requiring a greater number of endorsements in strictly defined cases than in liberally defined cases. Liberally defined cases increase the sample size, while strictly defined cases will reduce the rate of misclassification. We performed cross-trait PRS analyses, testing for association between PRSs for autoimmune diseases and depression, and vice versa. Motivated by the observation of sex-dependent genetic correlations between schizophrenia and autoimmune diseases (12), and by higher prevalence of both depression and autoimmune diseases in females, we stratified PRS analyses by sex. Our study is one of the largest to explore pleiotropy between depression and autoimmune diseases and elucidates the contribution of shared genetic influences to the observed comorbidity.

## Methods and Materials

### Participants

The UKB is a prospective health study of 500,000 individuals in the United Kingdom. Participants were identified through National Health Service patient registers if they were 40 to 69 years of age during the recruitment phase (2006–2010) and living in proximity to an assessment center. Participants attended a baseline assessment and contributed health information via touchscreen questionnaires and verbal interviews (13). Subsets of participants completed repeat assessments: instance 1 comprised *n =* 20,335 between 2012 and 2013; instance 2 comprised *n =* 42,961 (interview) and *n =* 48,340 (touchscreen) in 2014; and instance 3 comprised *n =* 2843 (interview) and *n =* 3081 (touchscreen) in 2019. Participant data are linked to Hospital Episode Statistics containing episodes of inpatient care. Episodes are coded at admission using the ICD-10 (14). Inpatients are assigned one primary code (reason for admission) and a variable number of secondary codes. Additional data are available for psychiatric phenotyping, including an online Mental Health Questionnaire completed by 157,366 participants in 2017 (15). The UKB received ethical approval from the North West–Haydock Research Ethics Committee (reference 16/NW/0274). Participants provided electronic signed consent at recruitment (13).

### Autoimmune Phenotyping

Guided by studies that investigated the epidemiological relationship between autoimmune diseases and depression (1,5) we identified cases for 14 autoimmune diseases: pernicious anemia (PA), autoimmune thyroid disease, type 1 diabetes (T1D), multiple sclerosis (MS), myasthenia gravis (MG), celiac disease, IBD (includes Crohn’s disease and ulcerative colitis), psoriasis, ankylosing spondylitis, polymyalgia rheumatica/giant cell arteritis, psoriatic arthritis (PsA), RA, Sjögren syndrome, and systemic lupus erythematosus (SLE).

Two sources of information were used to define autoimmune cases and controls: 1) Hospital Episode Statistics, in which primary and secondary ICD-10 diagnoses recorded between April 1997 to October 2016 were identified from the UKB Data Portal Record Repository; and 2) verbal interview, in which participants’ responses at baseline or instance 1 or 2 were used to determine self-endorsed medical conditions (past and current) and self-endorsed prescription medications (current). ICD-10 codes, self-endorsed conditions, and medications used to define each autoimmune disease are listed in Supplement 2.

**Figure 1:**
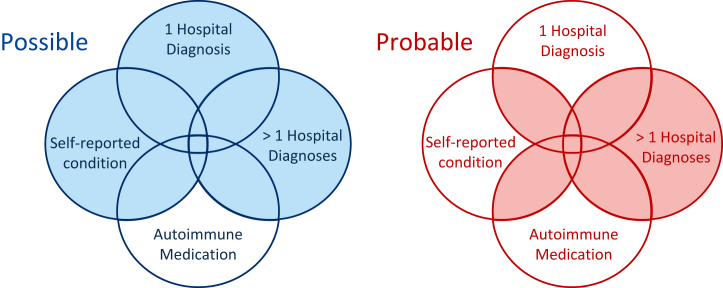
Autoimmune phenotyping approach. Cases are included in possible or probable if they fall within a shaded area. Autoimmune medication was used as a confirmatory but not as a primary source of information because several medications are not disease specific.

We took two approaches to defining autoimmune cases (Figure 1). To increase sample size, we created possible cases, comprising participants with an ICD-10 diagnosis or a self-endorsed condition. To increase validity, we used multiple criteria to define probable cases. Participants were coded as probable cases if at least two of ICD-10 diagnosis, self-endorsed condition, or medication were observed. More than one ICD-10 diagnosis for the corresponding autoimmune disease was also sufficient. A set of autoimmune controls was defined from participants with no ICD-10 diagnoses, self-endorsed conditions, or medications for all 14 autoimmune diseases. A single set of controls was used for all autoimmune diseases, given the known comorbidity between them.

### Depression Phenotyping

We created two depression case groups: strictly defined cases, termed “stringent depression,” and liberally defined cases, termed “any depression.” We have previously shown that single nucleotide polymorphism (SNP)–based heritability increases with multiple endorsements of depression (16). We classified stringent depression as participants endorsing at least three of the following depression measures: ICD-10 diagnoses (F32–F33.9), self-reported depression, self-reported antidepressant usage, single or recurrent depression [defined by Smith *et al.* (17) from responses to a questionnaire completed at baseline by 172,751 participants], or answering “yes” to the questionnaire: “Have you ever seen a GP/psychiatrist for nerves, anxiety, tension or depression?”

We classified “any depression” as participants who endorsed two or more depression measures, or if they met criteria for lifetime depression in the Composite International Diagnostic Interview assessed in the Mental Health Questionnaire (15). We classify cases defined from the Composite International Diagnostic Interview alone as “any depression” rather than as “stringent depression” because we previously observed lower SNP-based heritability in this group (*h*^2^_SNP_ = 11%, SE = 0.008) compared with cases defined by three or more non–Composite International Diagnostic Interview measures of depression (*h*^2^_SNP_ = 19%, SE = 0.018) (16).

Depression cases were screened for schizophrenia and bipolar according to any indication: ICD-10 diagnoses (F20–F29, F30–F31.9, F34–F39), self-endorsed conditions (schizophrenia, mania, bipolar disorder or manic depression) or self-endorsed antipsychotic usage reported at baseline or instance 1 or 2, bipolar type I (mania) or bipolar type II (hypomania) according to criteria adopted by Smith *et al.* (17), or indications of psychosis endorsed in the Mental Health Questionnaire. A single set of depression controls was defined from participants who did not meet the criteria for depression, schizophrenia, or bipolar disorder.

Derivation of depression, schizophrenia, and bipolar indications can be found in the Supplement from our previous publication (16).

### Genetic Quality Control

The UKB performed preliminary quality control (QC) on genotype data assayed for all participants (13). Using genetic principal components (PCs) provided by the UKB, we performed 4-means clustering on the first two PCs to identify and retain individuals of European ancestry. QC was then performed using PLINK v1.9 (www.cog-genomics.org/plink/1.9) (18) to remove variants with missingness >0.02 (before individual QC), individuals with missingness >0.02, individuals whose self-reported sex was discordant from their genetic sex, variants with missingness >0.02 (after individual QC), variants departing from Hardy-Weinberg equilibrium (*p <* 10^−8^), and variants with minor allele frequency <0.01. Relatedness kinship estimates provided by the UKB were used to identify pairs of related individuals (KING *r*^2^ > .044) (19) and the GreedyRelated (20) algorithm used to remove 1 individual from each pair, preferentially retaining individuals that survived QC. FlashPCA2 (21) was used to generate PCs for the subset of individuals of European ancestry surviving QC. PRS analyses were performed using genotype data.

### Statistical Analyses

We summarized sociodemographic data taken at baseline assessment: age, sex, socioeconomic status, body mass index, and current smoking status. We tested for significant differences in sociodemographic variables between cases and controls using Welch 2-sample *t* tests in R version 3.6 (R Foundation for Statistical Computing, Vienna, Austria). We tested for significant differences in 1) the prevalence of depression in autoimmune cases compared with autoimmune controls and 2) the prevalence of autoimmune diseases in depression cases compared with depression controls. These tests were performed for both probable/possible autoimmune cases and stringent/any depression, using 2-sample tests for equality of proportions in R version 3.6.

### Summary Statistics for Autoimmune Diseases and Depression

We searched PubMed and the NHGRI-EBI GWAS Catalog (https://www.ebi.ac.uk/gwas/downloads/summary-statistics) for the latest genome-wide association study (GWAS) with publicly available summary statistics, using the name of the relevant trait (and *GWAS* or *genome-wide association study* on PubMed). We identified summary statistics for 8 of the 14 autoimmune diseases: celiac (22), IBD (23), MS (24), MG (25), psoriasis (26), PsA (27), RA (28), and SLE (29) (Table 1). For MG, psoriasis, and PsA, we contacted the authors of the primary GWASs to obtain access. Summary statistics from GWASs using the Immunochip (Illumina, San Diego, CA) were excluded, as it does not provide genome-wide coverage. For major depressive disorder (MDD), we used summary statistics from Wray *et al.* (11), excluding the UKB.

**Table 1:** Genome-wide Association Study Summary Statistics Used to Generate Polygenic Risk Scores

Trait	Year	Ancestry	Assembly	Cases	Controls
Celiac Disease	2010	European	GRCh37/hg19	4533	10,750
Inflammatory Bowel Disease	2015	European	GRCh37/hg19	12,882	21,770
Multiple Sclerosis	2011	European	GRCh37/hg19	9772	17,376
Myasthenia Gravis	2015	European	GRCh37/hg19	1032	1998
Psoriasis	2017	European	GRCh37/hg19	19,032	286,769
Psoriatic Arthritis	2018	European	GRCh37/hg19	1430	1417
Rheumatoid Arthritis	2014	European	GRCh37/hg19	14,361	43,923
System Lupus Erythematosus	2015	European	GRCh37/hg19	7219	15,991
Major Depressive Disorder	2018	European	GRCh37/hg19	116,404	314,990

### PRS Analyses

PRS analyses were conducted using the PRSice-2 software (30). QC was performed on summary statistics to remove variants within the major histocompatibility complex (28.8–33.7 Mb), and default clumping settings were applied in PRSice-2 to remove variants in linkage disequilibrium (*r*^2^ > .1) with the lead variant within a 250-kb region.

To validate our phenotyping approach, we tested for association between PRSs for 8 autoimmune diseases and case-control status for the corresponding diseases (possible and probable cases), and between PRSs for MDD and case-control status for depression (any and stringent).

To investigate pleiotropy between autoimmune diseases and depression, we performed cross-trait analyses, testing for association between 1) PRSs for 8 autoimmune diseases and case-control status for depression (any and stringent cases) and 2) PRSs for MDD and case-control status for 14 autoimmune diseases (possible and probable cases). To test for sex-specific effects, we performed cross-trait analyses in males and females separately.

For each test, PRSs constructed at eight *p-*value thresholds (*p*_T_) (.001, .05, .1, .2, .3, .4, .5, and 1.0) were regressed on case-control status using logistic regression, adjusting for the following covariates: six PCs, genotyping batch, and assessment center (*n* = 128 variables). We report *p* values at the optimal *p*_T_ for each test. To control for multiple testing across *p*_T_ (×8) and across tests of association (autoimmune PRSs [×8] predicting any/stringent depression [×2] in men and women [×2], *n* = 32; and MDD PRSs predicting possible/probable [×2] autoimmune diseases [×14] in men and women [×2], *n* = 56), a Bonferroni correction was applied to give a *p-*value threshold for significance of 7.1 × 10^−5^ [.05 / 704 tests, 704 = 8 × (32 + 56)]. Where sex-specific associations were observed, sensitivity analyses were conducted to account for different sample sizes between sexes. We tested for interactions between sex and PRSs (at the optimal *p*_T_ from sex-specific tests) in the full sample (phenotype ∼ sex + PRSs + [sex × PRSs] + covariates). We report *R*^2^ estimates transformed to the liability scale using the following population prevalences for outcome traits: PA = 0.1% (31), autoimmune thyroid disease = 2% (32), T1D = 0.3% (33), MS = 0.1% (24), MG = 0.02% (34), celiac = 1% (22), IBD = 0.5% (23), psoriasis = 2% (26), ankylosing spondylitis = 0.55% (35), polymyalgia rheumatica/giant cell arteritis = 0.85% (36), PsA = 0.5% (27), RA = 1% (37), Sjögren syndrome = 0.7% (38), SLE = 0.1% (39), and MDD = 15% (11).

AVENGEME (40) was used to estimate power to detect cross-trait PRS associations, assuming varying degrees of genetic correlation (*r*_G_) between corresponding traits (*r*_G_ = .01–.5). Power was estimated for cross-trait analyses in which summary statistics for both traits were available (i.e., 8 autoimmune disorders and MDD) so that SNP-based heritability (required for power calculations in AVENGEME) could be estimated using linkage disequilibrium score regression (LDSC) (v1.0.1) (41) (Figure S1 in Supplement 1). Power was estimated using PRSs at the optimal *p*_T_ identified in cross-trait association tests, and liberally defined sample sizes. Parameters used to estimate power are in Tables S1 and S2 in Supplement 1.

LDSC v1.0.1 (41) was used to estimate *r*_G_ between the UKB depression phenotypes (any and stringent) and autoimmune diseases with publicly available summary statistics. To robustly apply LDSC, we limited the autoimmune diseases to those with sample sizes above 5000 in the primary GWAS [celiac (22), IBD (23), MS (24), psoriasis (26), RA (28), and SLE (29)]. To control for multiple testing across traits, a Bonferroni correction was applied to give a *p-*value threshold for significance of 4.1 × 10^−3^ in *r*_G_ analyses (.05/12 tests).

## Results

A total of 28,479 individuals were identified as possible cases across 14 autoimmune diseases, and a subset of 16,824 (59.1%) met the stringent criteria for probable cases (refer to Supplement 2 for representation of the overlap between criteria used to define cases). A total of 65,075 individuals met the criteria for any depression, 14,625 of whom met the criteria for stringent depression. Sociodemographic characteristics for autoimmune and depression cases and controls are summarized in Table 2. Overall, autoimmune and depression case groups contained a higher proportion of females, had lower socioeconomic status, had higher smoking prevalence, and had higher body mass index than their respective control groups (*n =* 324,074 autoimmune controls, *n =* 232,552 depression controls; all *p* values <5 × 10^−21^ in pairwise comparisons).

**Table 2:** Sociodemographic Information for Autoimmune and Depression Cases and Controls

	Pop. Prev.	Count	UKB Prev.	Age, Years, Mean (SD)	Female	TDI, Mean (SD)	Current Smoker	BMI, kg/m^2^, Mean (SD)	Count	UKB Prev.	Age, Years, Mean (SD)	Female	TDI, Mean (SD)	Current Smoker	BMI, kg/m^2^, Mean (SD)
		Possible							Probable						
Autoimmune Diseases															
Circulatory System															
Pernicious anemia	0.10%	1555	0.48%	58.9 (7.61)	71%	−0.85 (3.19)	13%	28.0 (5.55)	423	0.13%	60.1 (7.19)	72%	−0.99 (3.23)	12%	28.5 (5.34)

Endocrine System															
Autoimmune thyroid disease	2.00%	859	0.26%	56.8 (7.75)	85%	−1.11 (3.05)	15%	27.4 (5.11)	607	0.19%	57.0 (7.64)	86%	−1.06 (3.03)	16%	27.8 (5.21)
Type 1 diabetes	0.30%	2751	0.84%	58.3 (7.80)	42%	−0.46 (3.39)	13%	30.1 (6.08)	2292	0.70%	58.0 (7.85)	43%	−0.50 (3.39)	12%	29.9 (6.07)

Nervous System															
Multiple sclerosis	0.10%	1683	0.52%	55.4 (7.52)	73%	−1.45 (3.01)	16%	26.9 (5.08)	1154	0.35%	55.5 (7.45)	73%	−1.33 (3.06)	17%	26.9 (5.27)
Myasthenia gravis	0.02%	234	0.07%	59.2 (7.39)	56%	−0.94 (3.27)	11%	29.0 (5.44)	147	0.05%	60.1 (7.09)	48%	−1.06 (3.32)	12%	29.3 (5.18)

Digestive System															
Celiac	1.00%	2364	0.72%	57.8 (7.79)	67%	−1.49 (2.99)	7%	25.8 (4.57)	1260	0.39%	58.4 (7.66)	68%	−1.42 (3.03)	7%	25.8 (4.60)
Inflammatory bowel disease	0.50%	5105	1.55%	57.4 (7.92)	51%	−1.27 (3.10)	10%	27.3 (4.70)	3538	1.08%	57.3 (7.98)	50%	−1.28 (3.07)	9%	27.2 (4.63)

Skin															
Psoriasis	2.00%	5459	1.66%	56.8 (8.01)	46%	−1.05 (3.22)	15%	28.4 (5.17)	2759	0.84%	57.1 (8.04)	42%	−0.90 (3.28)	16%	28.7 (5.26)

Musculoskeletal System and Connective Tissue															
Ankylosing spondylitis	0.55%	1344	0.41%	57.9 (7.47)	38%	−1.02 (3.21)	13%	27.8 (4.80)	413	0.13%	57.4 (7.57)	26%	−1.06 (3.22)	12%	27.9 (4.85)
Polymyalgia rheumatica/GCA	0.85%	1627	0.50%	63.1 (5.16)	67%	−1.70 (2.79)	9%	28.3 (5.08)	898	0.28%	63.7 (4.58)	68%	−1.67 (2.75)	9%	28.4 (5.24)
Psoriatic arthritis	0.50%	1107	0.34%	56.6 (7.48)	50%	−1.16 (3.18)	11%	29.0 (5.43)	779	0.24%	56.6 (7.52)	51%	−1.06 (3.23)	11%	29.3 (5.59)
Rheumatoid arthritis	1.00%	6360	1.92%	59.5 (7.06)	67%	−0.91 (3.29)	13%	28.5 (5.53)	3451	1.05%	59.6 (7.01)	70%	−1.08 (3.18)	12%	28.2 (5.50)
Sjögren syndrome	0.70%	647	0.20%	59.3 (7.10)	90%	−1.20 (3.03)	6%	27.1 (5.72)	389	0.12%	59.2 (7.26)	89%	−1.19 (3.10)	7%	27.0 (5.55)
Systemic lupus erythematosus	0.10%	624	0.19%	56.7 (8.18)	84%	−1.02 (3.21)	14%	27.3 (5.65)	362	0.11%	56.6 (8.00)	86%	−0.99 (3.15)	14%	27.3 (5.82)
Any Autoimmune Disease	NA	28,479	8.08%	58.1 (7.73)	58%	−1.11 (3.17)	12%	27.9 (5.28)	16,824	4.94%	58.1 (7.73)	57%	−1.10 (3.17)	12%	28.0 (5.34)
Autoimmune Controls	NA	324,074	NA	56.4 (8.06)	52%	−1.50 (2.98)	10%	27.2 (4.64)	324,074	NA	56.4 (8.06)	52%	−1.50 (2.98)	10%	27.2 (4.64)

Depression			Any						Stringent						
Depression Cases	15%	65,075	21.86%	55.4 (7.86)	67%	−1.09 (3.14)	13%	27.8 (5.32)	14,625	5.92%	56.1 (7.88)	67%	−0.58 (3.35)	19%	28.9 (5.77)
Depression Controls	NA	232,552	NA	57.1 (8.10)	47%	−1.65 (2.89)	9%	27.2 (4.53)	232,552	NA	57.1 (8.10)	47%	−1.65 (2.89)	9%	27.2 (4.53)

Negative scores on the TDI indicate less deprivation.

BMI, body mass index; GCA, giant cell arteritis; NA, not applicable; Pop. Prev., population prevalence estimate; TDI, Townsend deprivation index; UKB Prev., prevalence of cases in the UK Biobank as a proportion of autoimmune/depression controls.

The prevalence of any depression was significantly higher in autoimmune cases than in autoimmune controls (*p =* 6 × 10^−177^ for possible cases of any autoimmune disease vs. controls, *p =* 2 × 10^−124^ for probable cases of any autoimmune disease vs. controls). The prevalence of stringent depression was significantly higher in autoimmune cases than in autoimmune controls (*p =* 3 × 10^−207^ for possible cases of any autoimmune disease vs. controls, *p =* 6 × 10^−163^ for probable cases of any autoimmune disease vs. controls) (Table 3).

**Table 3:** Prevalence of Depression Within Autoimmune Cases Compared With Autoimmune Controls, Stratified by Possible/Probable for Autoimmune Diseases and Any/Stringent for Depression Cases

Autoimmune Trait	Depression Prevalence in Autoimmune Traits			
	Any Depression Prevalence		Stringent Depression Prevalence	
	Possible Autoimmune	Probable Autoimmune	Possible Autoimmune	Probable Autoimmune
Autoimmune Controls	20.6%		5.1%	

Any Autoimmune Disease	28.9% (6 × 10^−177^)	29.5% (2 × 10^−124^)	10.8% (3 × 10^−207^)	11.5% (6 × 10^−163^)

Circulatory System				
Pernicious anemia	35.8% (4 × 10^−36^)	33.7% (4 × 10^−8^)	15.2% (1 × 10^−39^)	16.6% (4 × 10^−15^)

Endocrine System				
Autoimmune thyroid disease	35.1% (6 × 10^−19^)	34.7% (6 × 10^−13^)	13.7% (1 × 10^−16^)	13.4% (3 × 10^−11^)
Type 1 diabetes	31.1% (3 × 10^−31^)	31.3% (1 × 10^−27^)	14.1% (5 × 10^−59^)	13.5% (5 × 10^−43^)

Nervous System				
Multiple sclerosis	39.3% (6 × 10^−56^)	42.5% (3 × 10^−51^)	16.9% (5 × 10^−54^)	20.0% (8 × 10^−57^)
Myasthenia gravis	33.3% (5 × 10^−5^)	29.2% (3 × 10^−2^)	14.1% (5 × 10^−6^)	14.8% (4 × 10^−5^)

Digestive System				
Celiac	27.3% (3 × 10^−12^)	29.8% (2 × 10^−12^)	8.4% (4 × 10^−8^)	9.5% (7 × 10^−8^)
Inflammatory bowel disease	24.9% (6 × 10^−11^)	24.9% (3 × 10^−8^)	8.9% (8 × 10^−22^)	9.1% (2 × 10^−17^)

Skin				
Psoriasis	26.8% (2 × 10^−22^)	28.3% (1 × 10^−17^)	8.7% (4 × 10^−20^)	9.9% (4 × 10^−18^)

Musculoskeletal System and Connective Tissue				
Ankylosing spondylitis	26.9% (5 × 10^−7^)	28.4% (5 × 10^−4^)	9.6% (7 × 10^−9^)	10.5% (9 × 10^−5^)
Polymyalgia rheumatica/GCA	29.3% (2 × 10^−13^)	27.8% (5 × 10^−6^)	10.8% (3 × 10^−15^)	10.6% (1 × 10^−8^)
Psoriatic arthritis	31.8% (3 × 10^−15^)	34.3% (5 × 10^−16^)	10.2% (1 × 10^−8^)	13.0% (2 × 10^−13^)
Rheumatoid arthritis	30.6% (5 × 10^−62^)	29.4% (6 × 10^−28^)	12.7% (3 × 10^−92^)	12.1% (1 × 10^−45^)
Sjögren syndrome	40.7% (4 × 10^−25^)	41.9% (1 × 10^−17^)	18.9% (2 × 10^−28^)	18.9% (7 × 10^−18^)
Systemic lupus erythematosus	40.2% (2 × 10^−25^)	44.5% (2 × 10^−22^)	18.8% (6 × 10^−30^)	22.2% (8 × 10^−27^)

The *p* values from pairwise comparisons of depression prevalence in autoimmune cases compared with autoimmune controls are shown in parentheses.

GCA, giant cell arteritis.

The prevalence of possible cases of any autoimmune disease was significantly higher in depression cases than in depression controls (*p =* 6 × 10^−177^ for any depression vs. controls, *p =* 3 × 10^−207^ for stringent depression vs. controls). The prevalence of probable cases of any autoimmune disease was significantly higher in depression cases than in depression controls (*p =* 2 × 10^−124^ for any depression vs. controls, *p =* 6 × 10^−163^ for stringent depression vs. controls) (Table 4).

**Table 4:** Prevalence of Autoimmune Diseases Within Depression Cases Compared With Depression Controls, Stratified by Possible/Probable for Autoimmune Diseases and Any/Stringent for Depression Cases

Autoimmune Trait	Autoimmune Prevalence in Depression					
	Possible Autoimmune Prevalence			Probable Autoimmune Prevalence		
	Any Depression	Stringent Depression	Depression Controls	Any Depression	Stringent Depression	Depression Controls
Any Autoimmune Disease	10.48% (6 × 10^−177^)	14.27% (3 × 10^−207^)	6.94%	6.59% (2 × 10^−124^)	9.48% (6 × 10^−163^)	4.19%

Circulatory System						
Pernicious anemia	0.77% (4 × 10^−36^)	1.17% (1 × 10^−39^)	0.36%	0.19% (4 × 10^−8^)	0.35% (4 × 10^−15^)	0.10%

Endocrine System						
Autoimmune thyroid disease	0.41% (6 × 10^−19^)	0.58% (1 × 10^−16^)	0.20%	0.28% (6 × 10^−13^)	0.39% (3 × 10^−11^)	0.14%
Type 1 diabetes	1.20% (3 × 10^−31^)	2.07% (5 × 10^−59^)	0.69%	1.02% (1 × 10^−27^)	1.65% (5 × 10^−43^)	0.58%

Nervous System						
Multiple sclerosis	0.87% (6 × 10^−56^)	1.31% (5 × 10^−54^)	0.35%	0.63% (3 × 10^−51^)	1.02% (8 × 10^−57^)	0.22%
Myasthenia gravis	0.11% (5 × 10^−5^)	0.17% (5 × 10^−6^)	0.05%	0.06% (3 × 10^−2^)	0.12% (4 × 10^−5^)	0.04%

Digestive System						
Celiac	0.92% (3 × 10^−12^)	1.08% (4 × 10^−8^)	0.64%	0.54% (2 × 10^−12^)	0.64% (7 × 10^−8^)	0.33%
Inflammatory bowel disease	1.82% (6 × 10^−11^)	2.56% (8 × 10^−22^)	1.43%	1.28% (3 × 10^−8^)	1.85% (2 × 10^−17^)	1.00%

Skin						
Psoriasis	2.05% (2 × 10^−22^)	2.55% (4 × 10^−20^)	1.46%	1.10% (1 × 10^−17^)	1.47% (4 × 10^−18^)	0.73%

Musculoskeletal System and Connective Tissue						
Ankylosing spondylitis	0.52% (5 × 10^−7^)	0.72% (7 × 10^−9^)	0.37%	0.17% (5 × 10^−4^)	0.25% (9 × 10^−5^)	0.11%
Polymyalgia rheumatica/GCA	0.66% (2 × 10^−13^)	0.93% (3 × 10^−15^)	0.42%	0.35% (5 × 10^−6^)	0.51% (1 × 10^−8^)	0.23%
Psoriatic arthritis	0.50% (3 × 10^−15^)	0.58% (1 × 10^−8^)	0.28%	0.38% (5 × 10^−16^)	0.51% (2 × 10^−13^)	0.19%
Rheumatoid arthritis	2.66% (5 × 10^−62^)	4.14% (3 × 10^−92^)	1.59%	1.42% (6 × 10^−28^)	2.23% (1 × 10^−45^)	0.89%
Sjögren syndrome	0.34% (4 × 10^−25^)	0.55% (2 × 10^−28^)	0.13%	0.21% (1 × 10^−17^)	0.33% (7 × 10^−18^)	0.08%
Systemic lupus erythematosus	0.36% (2 × 10^−25^)	0.59% (6 × 10^−30^)	0.14%	0.23% (2 × 10^−22^)	0.39% (8 × 10^−27^)	0.08%

The *p* values from pairwise comparisons of autoimmune prevalence in depression cases compared with depression controls are shown in parentheses.

**Figure 2:**
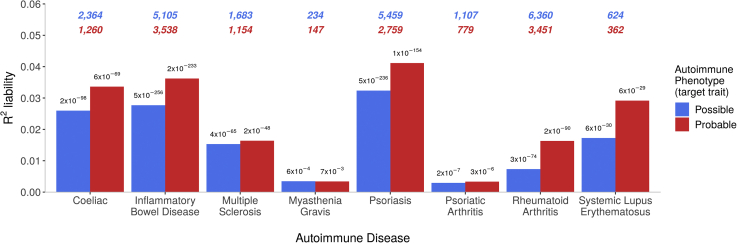
Variances in autoimmune liability explained by polygenic risk scores for the corresponding autoimmune diseases. The number of cases are shown at the top of the plot (possible = blue, probable = red). The *p* values are shown atop each bar.

Testing for same-trait PRS associations, PRSs for MDD were significantly associated with any depression case status (*p <* 5 × 10^−324^, *R*^2^ = 1.48%) and stringent depression case status (*p =* 2 × 10^−228^, *R*^2^ = 2.23%). PRSs for autoimmune diseases were significantly associated with both possible and probable case-control status for the corresponding diseases (Figure 2). The variance in liability, *R*^2^, explained by PRSs was higher in strictly defined compared with liberally defined phenotypes. Most results were highly significant (*p <* 6 × 10^−29^), except for MG (*p <* 7 × 10^−3^), which had the smallest sample size of 234 cases, and PsA (*p <* 3 × 10^−6^), in which the discovery GWAS had only 1430 cases.

Power analyses showed that in the prediction of any depression from autoimmune PRSs, there was 80% power to detect associations assuming modest levels of underlying genetic correlation (*r*_G_); *r*_G_ < .05 for celiac, MS, psoriasis, and SLE; *r*_G_ < .1 for IBD, PsA, and RA; and *r*_G_ < .17 for MG (Figure S2 in Supplement 1). In the prediction of possible autoimmune diseases from depression PRSs, there was 80% power to detect associations assuming *r*_G_ < .05 for celiac and IBD, *r*_G_ < .1 for psoriasis and RA, and *r*_G_ < .15 for PsA and SLE. There were two exceptions, MS and MG, in which the underlying *r*_G_ would need to approach ∼.3 to achieve 80% power (Figure S3 in Supplement 1).

**Figure 3:**
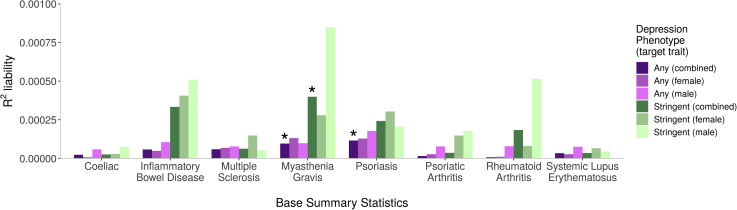
Variances in depression liability explained by polygenic risk score for autoimmune diseases (x-axis). Asterisks denote associations with *p* values <7.1 × 10^−5^, meeting Bonferroni correction. Number of cases for depression phenotypes: any (combined) = 65,075; any (female) = 43,413; any (male) = 21,662; stringent (combined) = 14,625; stringent (female) = 9738; stringent (male) = 4887.

In the prediction of depression from autoimmune PRSs (Figure 3), PRSs for MG were significantly associated with case status for any depression (*p =* 5.2 × 10^−5^, *R*^2^ = 0.01%) and stringent depression (*p =* 1.6 × 10^−5^, *R*^2^ = 0.04%). PRSs for psoriasis were significantly associated with case status for any depression (*p =* 8.7 × 10^−6^, *R*^2^ = 0.01%). No other autoimmune PRSs predicted depression case-control status, and no sex-specific analyses met the Bonferroni-corrected threshold. The *R*^2^ values for variance explained in depression by autoimmune PRSs were all very low, at <0.1%, and substantially lower than the *R*^2^ for autoimmune diseases (Figure 2).

**Figure 4:**
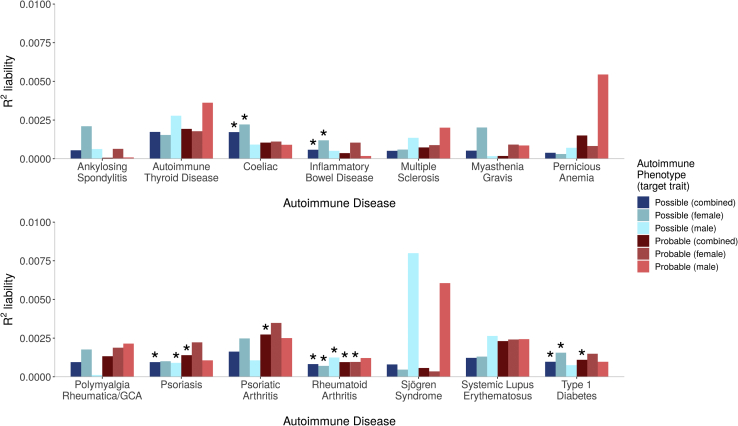
Variances in autoimmune liability (x-axes) explained by polygenic risk scores for major depressive disorder. Asterisks denote associations with *p* values <7.1 × 10^−5^, meeting Bonferroni correction. Number of cases for the autoimmune diseases are given in Table 2. GCA, giant cell arteritis.

In the prediction of autoimmune diseases from depression PRSs, genetic liability for MDD was significantly associated with six autoimmune diseases: celiac, IBD, psoriasis, PsA, RA, and T1D (all *p* values <5.8 × 10^−5^, *R*^2^ range between 0.06% and 0.27%) (Figure 4). For three, the association with MDD was observed in probable and possible cases (psoriasis, RA, and T1D). For celiac and IBD, the association was only in possible cases. For PsA, the association was only in probable cases. For all significant associations, higher PRSs increased risk for the outcome phenotype, except for celiac, in which higher MDD PRSs were associated with reduced risk (*p =* 6 × 10^−8^, *R*^2^ = 0.17%, beta = −0.11, SE = 0.02, in the combined sample).

In the prediction of autoimmune diseases from depression PRSs, sex-specific associations were observed, primarily in female autoimmune cases (celiac, IBD, T1D, and RA, all *p <* 4.5 × 10^−5^). Association in males was observed in psoriasis (possible cases, *p =* 5.8 × 10^−5^) and in RA (possible cases, *p =* 1.6 × 10^−5^). The most consistent results were observed in RA, in which the sample size was largest, with five of the six analyses reaching Bonferroni threshold (all *p <* 4.5 × 10^−5^, *R*^2^ range between 0.07% and 0.1%). However, there was no evidence for a significant interaction between sex and PRSs in the combined samples of men and women (all *p* > .02), indicating that sex-specific associations were generally influenced by sample size.

Full results of each test are shown in Tables S3 to S6 and Figures S4 to S7 in Supplement 1.

Significant genetic correlations (*r*_G_) were observed between IBD and any depression (*r*_G_ = .11; 95% confidence interval, .03–.18; *p =* 3.8 × 10^−3^) and stringent depression (*r*_G_ = .16; 95% confidence interval, .07–.24; *p =* 3.0 × 10^−4^) and between psoriasis and stringent depression (*r*_G_ = .16; 95% confidence interval, .06*–*.26; *p =* 1.1 × 10^−3^). No other traits met the Bonferroni-corrected threshold for significance in *r*_G_ analyses (Table S7 in Supplement 1).

## Discussion

Motivated by epidemiological findings of a bidirectional relationship between depression and autoimmune diseases, we tested for evidence of pleiotropy between these traits, adopting both liberal and strict phenotyping to define cases in the UKB. We showed modest association of PRSs from autoimmune diseases with MDD, and slightly stronger associations of MDD PRSs with autoimmune diseases. These observations suggest that only a minor component of observed comorbidity is due to shared genetics between depression and autoimmune diseases.

We made three key observations: 1) phenotypic variance explained by PRSs for corresponding traits was higher in strictly defined than liberally defined cases, indicating that more rigorous phenotyping improved the validity of autoimmune and depression cases; 2) the phenotypic overlap between depression and autoimmune diseases was consistent with the literature, in which depression was more common in individuals with autoimmune diseases, and vice versa; and 3) cross-trait PRS analyses identified significant associations between depression and some autoimmune diseases, but with effect sizes indicating the existence of a shared biological component of modest effect on the observed comorbidity.

Our phenotyping approach used both strictly defined and liberally defined cases, integrating the multiple sources of UKB data. PRSs for 8 autoimmune diseases predicted case-control status, increasing confidence in the robustness of case definition. The phenotypic variance explained was higher in strictly defined cases, potentially reflecting greater specificity; identifying individuals with multiple endorsements for a disease reduces the probability of misclassifying controls as cases. Conversely, the criteria for liberally defined cases increases sample size but may induce misclassification.

For each of the autoimmune diseases considered, cases had higher frequencies of depression than controls, recapitulating the effect observed in epidemiological studies. Similarly, the prevalence of each autoimmune disease was significantly higher in depression cases than in controls. Prevalence estimates reported here are cross-sectional, and we lack information on the temporal relationship between traits.

Cross-trait PRS analyses identified significant associations, although observed effect sizes were small, ranging between *R*^2^ = 0.01% and 0.27%. Compared with the substantially higher phenotypic variance explained by PRSs in corresponding traits, the small effect sizes observed in cross-trait PRS analyses provide a useful contrast, indicating only a small contribution of shared genetic influences in the observed comorbidities. However, this was not universally true—MDD PRSs captured nearly the same amount of variance in probable PsA (0.27%) as the PRSs for PsA (0.29%). For all significant associations, higher PRSs increased risk for the outcome phenotype. Interestingly, there was one exception, in which higher MDD PRSs were associated with reduced risk for celiac disease. This is intriguing, given the positive phenotypic correlation between depression and celiac disease, and may warrant further investigation.

For three traits, we observed significant associations in liberally defined but not strictly defined cases (psoriasis PRSs were associated with any depression, and MDD PRSs were associated with possible celiac and IBD). In contrast, MDD PRSs were associated with probable, but not possible, PsA, suggesting misclassification in possible cases. Misclassification bias may vary across diseases; some autoimmune diseases may be more prone to misclassification with other autoimmune diseases, while other diagnoses may misclassify with noninflammatory conditions. For example, osteoarthritis (noninflammatory) may misclassify as PsA in the absence of multiple-item endorsement to increase diagnosis validity.

Cross-trait PRS analyses identified some sex-dependent associations. MDD PRSs were associated with psoriasis in males and MDD PRSs were associated with celiac, IBD, and T1D in females. However, sensitivity analyses revealed no evidence for significant interactions between PRSs and sex in the combined sample, indicating that sex-dependent associations were generally driven by different sample sizes in sex-stratified analyses. RA was the most common autoimmune disease and showed the most consistency in cross-trait associations; MDD PRSs were significantly associated with RA in all case groups, except for probable males. This is in contrast with Euesden *et al.*(10), who found no evidence for association between depression PRSs and risk for RA, but in a smaller sample of 226 cases. Liu *et al.* (6) also found no evidence for association between composite mental health disorder PRSs and risk for autoimmune diseases, but also in a smaller sample of 1383 individuals with any of seven autoimmune diseases. However, composite PRSs for autoimmune diseases did show weak association with case-control status in a sample of 43,902 individuals with any of six mental health disorders. This highlights the importance of sample size, and our study benefits from the scale of the UKB, in which power calculations indicated our investigation was able to detect modest pleiotropic effects.

In contrast to the small, but significant, cross-trait PRS associations observed between depression and several autoimmune diseases, we only observed significant genetic correlations between depression and two autoimmune diseases: IBD and psoriasis. The PRS methodology, which exploits the use of individual-level data, may have increased power to detect weak genetic effects compared with LDSC, which uses only summary statistics.

The weak genetic contribution suggests that another mechanism may be driving or contributing to the bidirectional relationship between autoimmune diseases and depression (42). Inflammatory factors underlying some cases of depression could provide a common biological pathogenesis with autoimmune diseases. Lynall *et al.* (43) observed increased immune cell counts in depression cases compared with controls, and identified a subgroup of cases with elevated inflammatory markers who presented with more severe depression than uninflamed cases. Environmental risk factors such as body mass index and childhood maltreatment increase risk of both depression and autoimmune diseases and would contribute to the bidirectional effect (44,45). Similarly, some treatments for depression (antidepressants) and autoimmune diseases (steroids) are obesogenic and may increase comorbidity. Diagnosis with autoimmune disease increases risk of depression due to psychological factors in adjusting to a chronic disorder and changes in behavior such as reduced exercise. Health-related behaviors that are elevated in depression (smoking, poor diet, reduced physical activity) increase risk for autoimmune diseases. These mechanisms may not be independent of joint genetic contributors. For example, shared inflammatory mechanisms would lead to horizontal pleiotropy, in which genetic variants directly affect both disorders, and vertical pleiotropy can arise through environmental risk factors in which genetic variation influences one trait through mediation on another trait. The mechanisms underpinning the observed cross-trait PRS associations may warrant further investigation, potentially using Mendelian randomization to investigate whether MDD risk alleles have a causal effect on autoimmune diseases, and vice versa. It is also interesting to speculate that associations could be driven by phenotypic hitchhiking, in which a GWAS for one trait (e.g., MDD) ascertains patients with comorbid diseases (e.g., autoimmune), potentially inducing cross-trait correlations. Disentangling pleiotropy from phenotypic hitchhiking may warrant further investigation.

### Limitations

A healthy volunteer bias has been observed in the UKB (46) and is a noted limitation of the study. However, it has been proposed that this bias may attenuate, but not invalidate, exposure-outcome relationships (47). A further limitation of the ability to extrapolate our results is the lack of representation in individuals of diverse ancestries. The literature has demonstrated attenuation in PRS analyses in which training and target samples come from different ancestral populations (48), highlighting the need to perform GWASs in diverse ancestries. This limitation may have broader implications than would otherwise be the case for some conditions, such as SLE, that disproportionately affect individuals of African and Asian ancestry.

Although every effort has been made to address the potential for misclassification through the criteria for multiple-item endorsements in strictly defined cases, the approach remains imperfect. For example, limited sample size led us to combine thyroiditis and Graves’ disease, which have opposing thyroid function, under the broader classification of autoimmune thyroid disease.

Power calculations showed that for some rare autoimmune diseases, larger samples would be required to reject the presence of a weak genetic correlation with depression. We also observed low SNP-based heritability using published summary statistics for MS, which reduced power to detect pleiotropic effects. The Bonferroni correction applied to cross-trait PRS analyses was conservative because the eight PRS *p* value thresholds included in each test of association are correlated, although it is difficult to determine the appropriate correction, and we chose to be strict rather than liberal.

### Conclusions

We identified cases and controls for depression and 14 autoimmune diseases in the UKB, using both strict and liberal phenotyping. PRS analyses indicated that strict phenotyping improved the validity of cases, demonstrating that multiple UKB variables can be leveraged to increase specificity. Consistent with the literature, we found that depression was enriched in autoimmune cases, and vice versa. Despite having power to detect subtle pleiotropic effects, we found little evidence that shared genetic factors have a meaningful influence on the observed co-occurrence of depression and autoimmune diseases in the UK Biobank. The limited shared genetic component will make only a modest contribution to the bidirectional disease risks, and shared environmental factors, including health-related characteristics and stressful life events, may be important. Future studies leveraging phenotypic, genetic, diagnostic, treatment and environmental risk factors may be necessary to unpick the mechanisms contributing to shared risks for autoimmune diseases and depression. In particular, future research should consider the psychological impacts of autoimmune disease while remaining cognizant of the need to consider and treat the two diseases in parallel.
