# Supplementary material for: Investigating Pleiotropy Between Depression and Autoimmune Diseases Using the UK Biobank
Source: Biol Psychiatry Glob Open Sci. 2021 Mar 25;1(1):48–58. doi: 10.1016/j.bpsgos.2021.03.002 (PMC8262258; doi:10.1016/j.bpsgos.2021.03.002)
Supplement: Supplement 1 [file mmc1.pdf]

Investigating Pleiotropy Between Depression and Autoimmune Diseases Using the UK Biobank

Supplement 1

Table of Contents

1 Power calculations 3

1.1 SNP-based heritability estimates 3

Supplementary Figure S1: SNP-based heritability (h^2_{SNP}) of traits with publicly available summary statistics. 3

1.2 Power estimates for autoimmune PRS predicting any depression in the UKB..........4

Supplementary Table S1: AVENGEME inputs for calculating power to detect association between PRS for autoimmune diseases and 'any' depression case status in the UKB..........4

Supplementary Figure S2: Power to detect associations between autoimmune PRS and any depression case status in UKB across different levels of genetic correlation (rG)..........5

1.3 Power estimates for depression PRS predicting autoimmune disease in the UKB..........6

Supplementary Table S2: AVENGEME inputs for calculating power to detect association between PRS for Major Depressive Disorder (MDD) and possible autoimmune diseases in UKB..........6

Supplementary Figure S3: Power to detect associations between MDD PRS and possible autoimmune case status in UKB across different levels of genetic correlation (rG)..........7

2 Polygenic risk score analyses 8

Supplementary Table S3: Within-trait PRS - associations between PRS for autoimmune diseases and autoimmune case/control status (possible/probable) at optimal P_T..........8

Supplementary Figure S4: Within-trait PRS - associations between PRS for autoimmune diseases and autoimmune case/control status (possible/probable) across eight P_T. 10

Supplementary Table S4: Within-trait PRS - associations between PRS for MDD and depression case/control status (any/stringent) at optimal P_T..........11

Supplementary Figure S5: Within-trait PRS - associations between PRS for MDD and depression case/control status (any/stringent) across eight P_T..........11

Supplementary Table S5: Cross-trait PRS - associations between PRS for autoimmune diseases and depression case/control status (any/stringent) in men and women at optimal P_T. 12

Supplementary Figure S6: Cross-trait PRS –associations between PRS for autoimmune diseases and depression case/control status (any/stringent) in men and women across eight P_T..........14

Supplementary Table S6: Cross-trait PRS - associations between PRS for MDD and autoimmune case/control status (possible/probable) in men and women at optimal P_T..........15

Supplementary Figure S7: Cross-trait PRS –associations between PRS for MDD and autoimmune case/control status (possible/probable) in men and women across eight P_T..........21

3 Genetic correlations..........22

Supplementary Table S7: Genetic correlations between autoimmune traits and ‘any’ and ‘stringent’ depression in the UKB. 22

4 References .... *Error! Bookmark not defined.*

1 Power calculations

1.1 SNP-based heritability estimates

Estimates of SNP-based heritability are required for power calculations using the AVENGEME software. We estimated SNP-based heritability for depression and the eight autoimmune diseases for which summary statistics were publicly available (summary statistics described in Table 1 of the main paper), and the results are shown in Supplementary Figure 1.

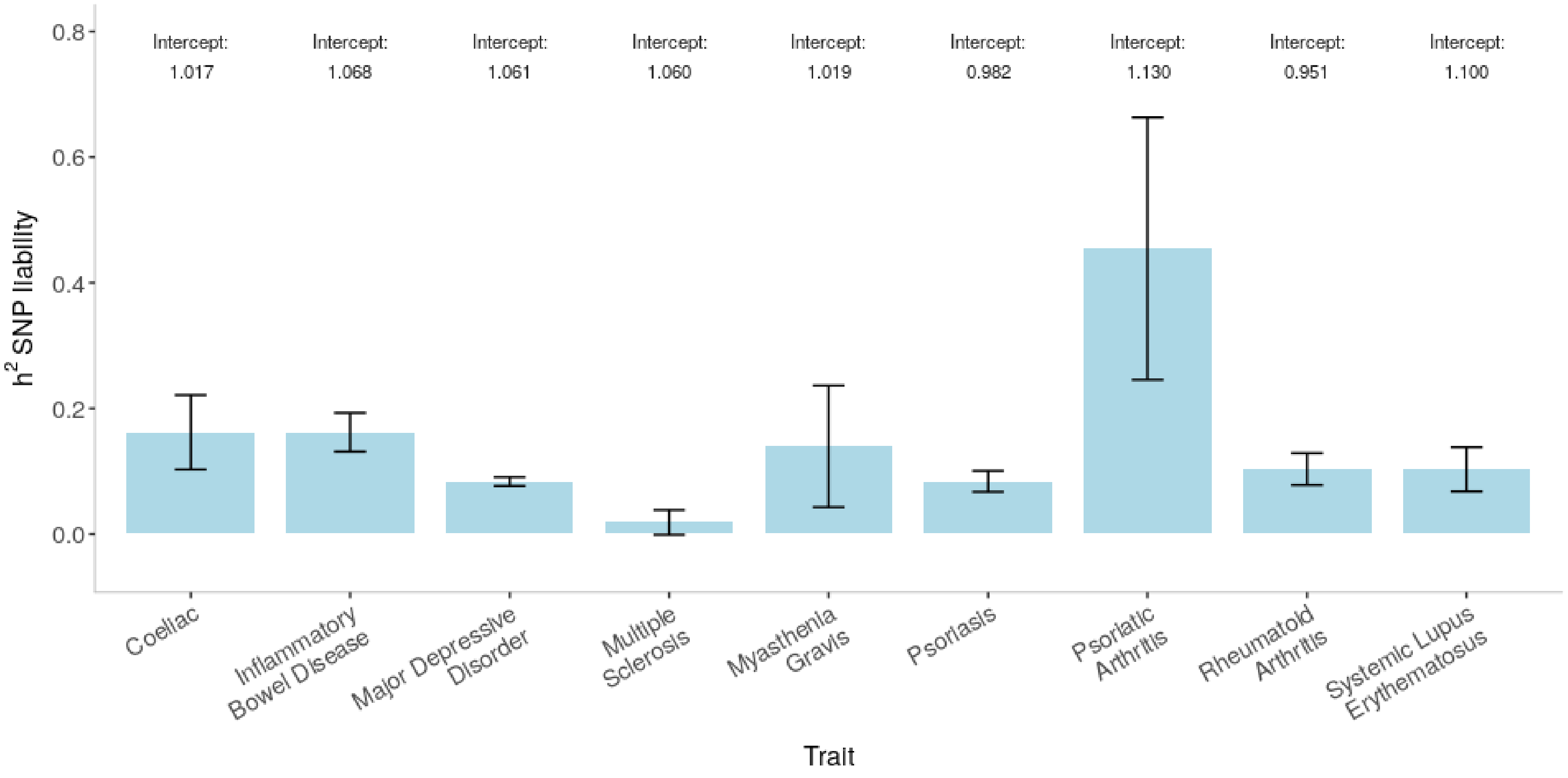

Supplementary Figure S1: SNP-based heritability (h^2_{SNP}) of traits with publicly available summary statistics. Estimated using LDSC after removing variants within the MHC from summary statistics. Transformed to liability scale using population prevalence estimates: Coeliac=1%, IBD=0.5%, MDD=15%, MS=0.1%, MG=0.02%, Psoriasis=2%, PsA=0.5%, RA=1%, SLE=0.1%. Error bars: 95% CIs. Intercepts shown atop bars.

1.2 Power estimates for autoimmune PRS predicting any depression in the UKB

Supplementary Table S1: AVENGEME inputs for calculating power to detect association between PRS for autoimmune diseases and 'any' depression case status in the UKB.

BASE GWAS							TARGET TRAIT				
Trait	Total N (case+control)	h^2_{SNP} excluding MHC	Population prevalence	Sample prevalence	Optimal p-value threshold (P_T)	No. SNPs used to construct PRS at (P_T)	Trait	Total N (case+control)	h^2_{SNP} excluding MHC	Population prevalence	Sample prevalence
Coeliac	15,283	16.21%	1.00%	30%	0.200	12,430	Any Depression	297,627	8.38%	15%	22%
Inflammatory Bowel Disease	34,652	16.20%	0.50%	37%	1.000	80,201	Any Depression	297,627	8.38%	15%	22%
Multiple Sclerosis	27,148	1.85%	0.10%	36%	0.050	3,240	Any Depression	297,627	8.38%	15%	22%
Myasthenia Gravis	3,030	13.96%	0.02%	34%	0.300	56,894	Any Depression	297,627	8.38%	15%	22%
Psoriasis	305,801	8.38%	2.00%	6%	1.000	136,084	Any Depression	297,627	8.38%	15%	22%
Psoriatic Arthritis	2,847	45.44%	0.50%	50%	0.050	4,666	Any Depression	297,627	8.38%	15%	22%
Rheumatoid Arthritis	58,284	10.35%	1.00%	25%	1.000	118,649	Any Depression	297,627	8.38%	15%	22%
Systemic Lupus Erythematosus	23,210	10.30%	0.10%	31%	0.001	836	Any Depression	297,627	8.38%	15%	22%

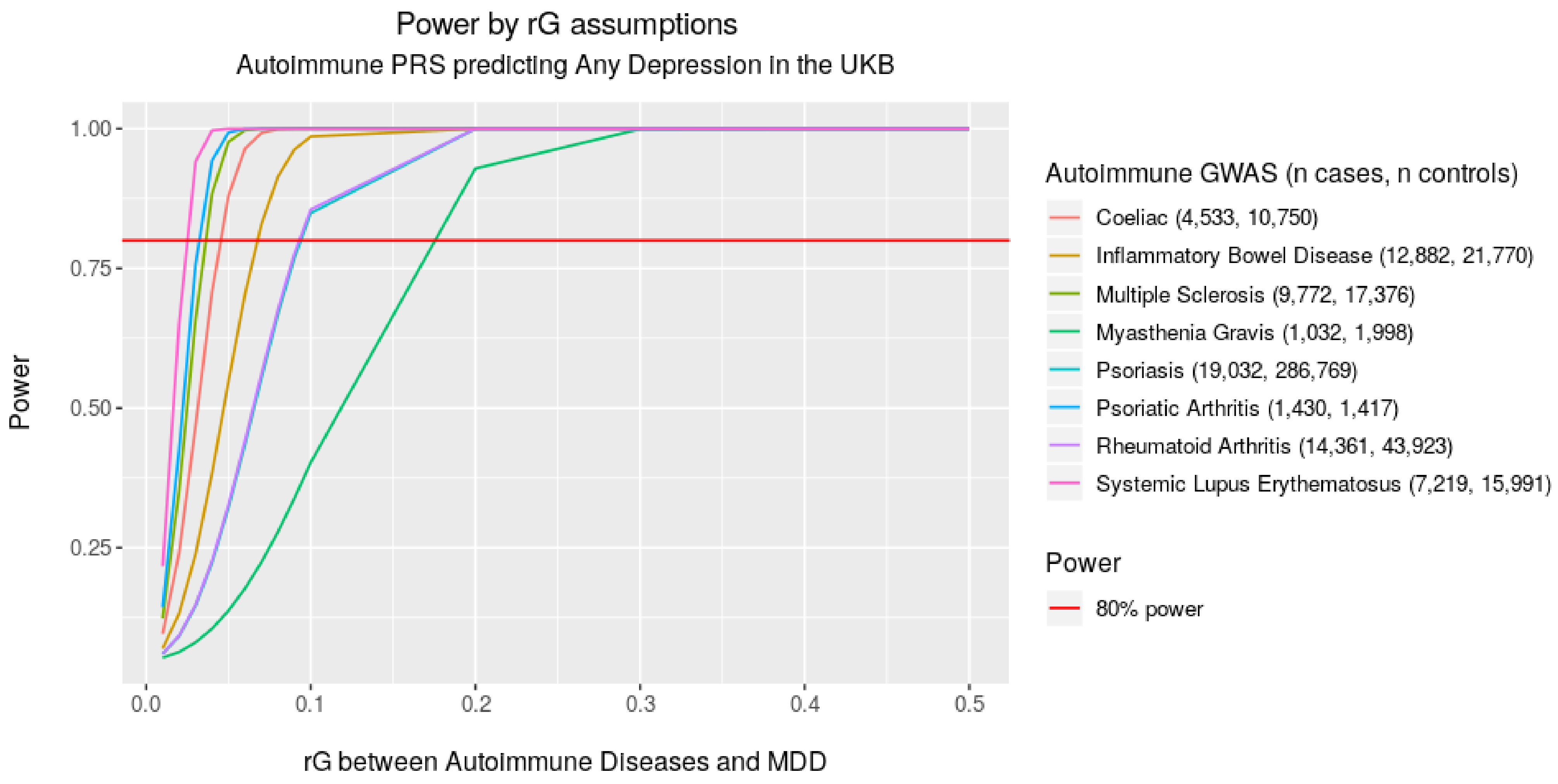

Supplementary Figure S2: Power to detect associations between autoimmune PRS and any depression case status in UKB across different levels of genetic correlation (rG).

1.3 Power estimates for depression PRS predicting autoimmune disease in the UKB

Supplementary Table S2: AVENGEME inputs for calculating power to detect association between PRS for Major Depressive Disorder (MDD) and possible autoimmune diseases in UKB.

BASE GWAS							TARGET TRAIT				
Trait	Total N (case+control)	h^2_{SNP} excluding MHC	Population prevalence	Sample prevalence	Optimal p-value threshold (P_T)	No. SNPs used to construct PRS at (P_T)	Trait	Total N (case+control)	h^2_{SNP} excluding MHC	Population prevalence	Sample prevalence
MDD	431,394	8.38%	15%	27%	0.001	1,123	Coeliac	326,438	16.21%	1.00%	0.72%
MDD	431,394	8.38%	15%	27%	0.300	48,852	Inflammatory Bowel Disease	329,179	16.20%	0.50%	1.55%
MDD	431,394	8.38%	15%	27%	0.500	67,956	Multiple Sclerosis	325,757	1.85%	0.10%	0.52%
MDD	431,394	8.38%	15%	27%	1.000	99,216	Myasthenia Gravis	324,308	13.96%	0.02%	0.07%
MDD	431,394	8.38%	15%	27%	0.500	67,956	Psoriasis	329,533	8.38%	2.00%	1.66%
MDD	431,394	8.38%	15%	27%	1.000	99,216	Psoriatic Arthritis	325,181	45.44%	0.50%	0.34%
MDD	431,394	8.38%	15%	27%	0.500	67,956	Rheumatoid Arthritis	330,434	10.35%	1.00%	1.92%
MDD	431,394	8.38%	15%	27%	0.050	14,137	Systemic Lupus Erythematosus	324,698	10.30%	0.10%	0.19%

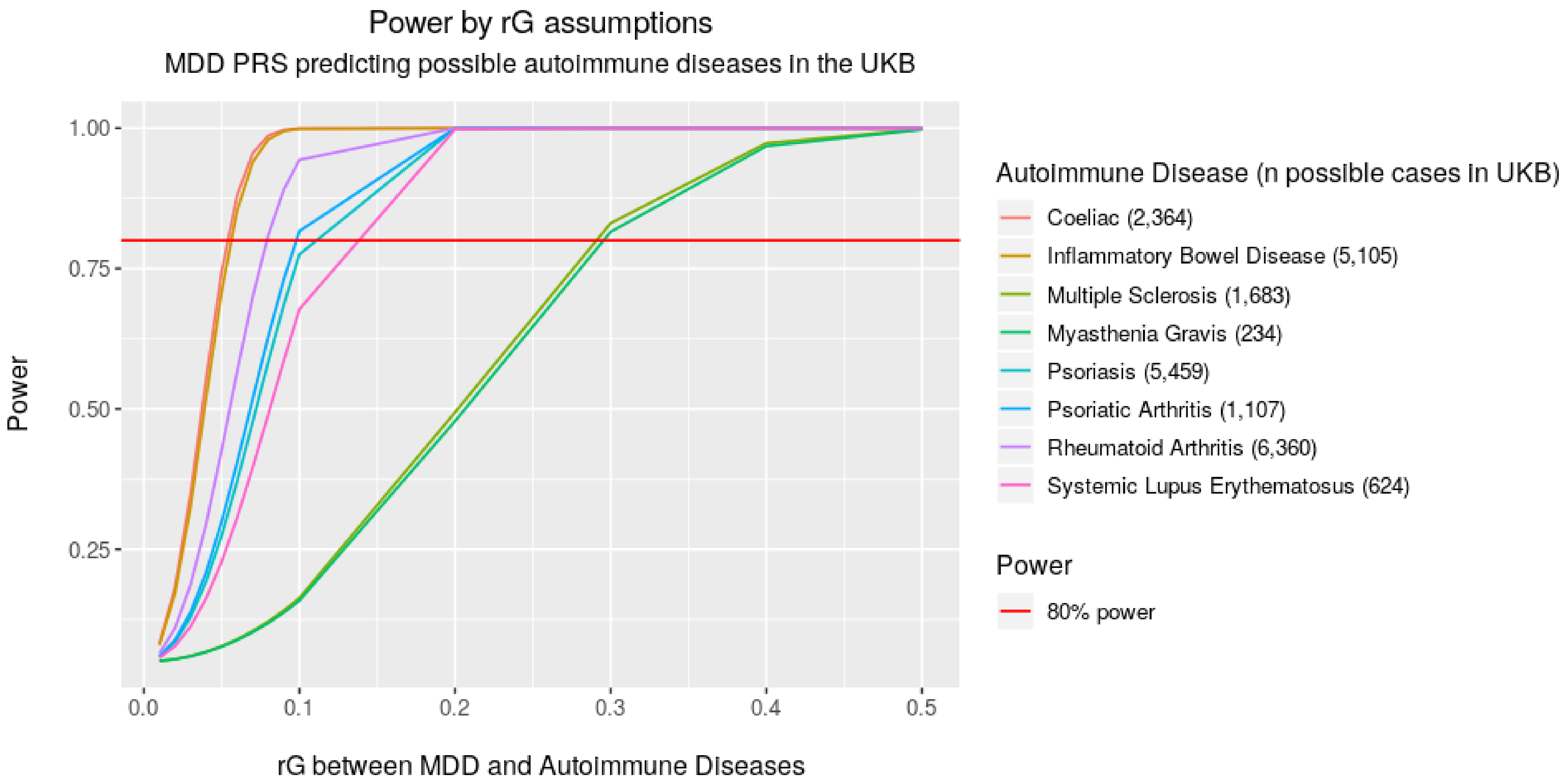

Supplementary Figure S3: Power to detect associations between MDD PRS and possible autoimmune case status in UKB across different levels of genetic correlation (rG).

2 Polygenic risk score analyses

Supplementary Table S3: Within-trait PRS - associations between PRS for autoimmune diseases and autoimmune case/control status (possible/probable) at optimal P_T.

Base GWAS	Target Trait in UKB	Optimal p-value threshold (P_T)	No. SNPs used to construct PRS at (P_T)	Population Prevalence used to convert to liability R^2	Unadjusted Variance explained (Observed R^2)	Adjusted Variance explained (Liability R^2)	Coefficient	Standard Error	P-value
Coeliac	Coeliac (Possible)	1.000	42,731	1.00%	1.65%	2.60%	0.46	0.022	2e-98
Coeliac	Coeliac (Probable)	1.000	42,731	1.00%	1.91%	3.37%	0.52	0.030	6e-69
Inflammatory Bowel Disease	Inflammatory Bowel Disease (Possible)	0.001	676	0.50%	2.41%	2.77%	0.49	0.014	5e-256
Inflammatory Bowel Disease	Inflammatory Bowel Disease (Probable)	0.001	676	0.50%	2.90%	3.62%	0.56	0.017	2e-233
Multiple Sclerosis	Multiple Sclerosis (Possible)	0.001	230	0.10%	1.42%	1.53%	0.42	0.025	4e-65
Multiple Sclerosis	Multiple Sclerosis (Probable)	0.001	230	0.10%	1.43%	1.64%	0.43	0.030	2e-48
Myasthenia Gravis	Myasthenia Gravis (Possible)	0.100	22,895	0.02%	0.31%	0.35%	0.23	0.066	6e-04
Myasthenia Gravis	Myasthenia Gravis (Probable)	0.100	22,895	0.02%	0.28%	0.34%	0.23	0.084	7e-03
Psoriasis	Psoriasis (Possible)	0.001	637	2.00%	2.08%	3.24%	0.44	0.013	5e-236
Psoriasis	Psoriasis (Probable)	0.001	637	2.00%	2.27%	4.11%	0.49	0.019	1e-154
Psoriatic Arthritis	Psoriatic Arthritis (Possible)	0.200	14,198	0.50%	0.19%	0.29%	0.27	0.052	2e-07
Psoriatic Arthritis	Psoriatic Arthritis (Probable)	1.000	45,914	0.50%	0.20%	0.34%	0.31	0.066	3e-06
Rheumatoid Arthritis	Rheumatoid Arthritis (Possible)	0.001	550	1.00%	0.58%	0.73%	0.23	0.013	3e-74
Rheumatoid Arthritis	Rheumatoid Arthritis (Probable)	0.001	550	1.00%	1.12%	1.63%	0.34	0.017	2e-90
Systemic Lupus Erythematosus	Systemic Lupus Erythematosus (Possible)	0.001	836	0.10%	1.37%	1.73%	0.42	0.037	6e-30
Systemic Lupus Erythematosus	Systemic Lupus Erythematosus (Probable)	0.300	74,372	0.10%	2.14%	2.92%	0.58	0.052	6e-29

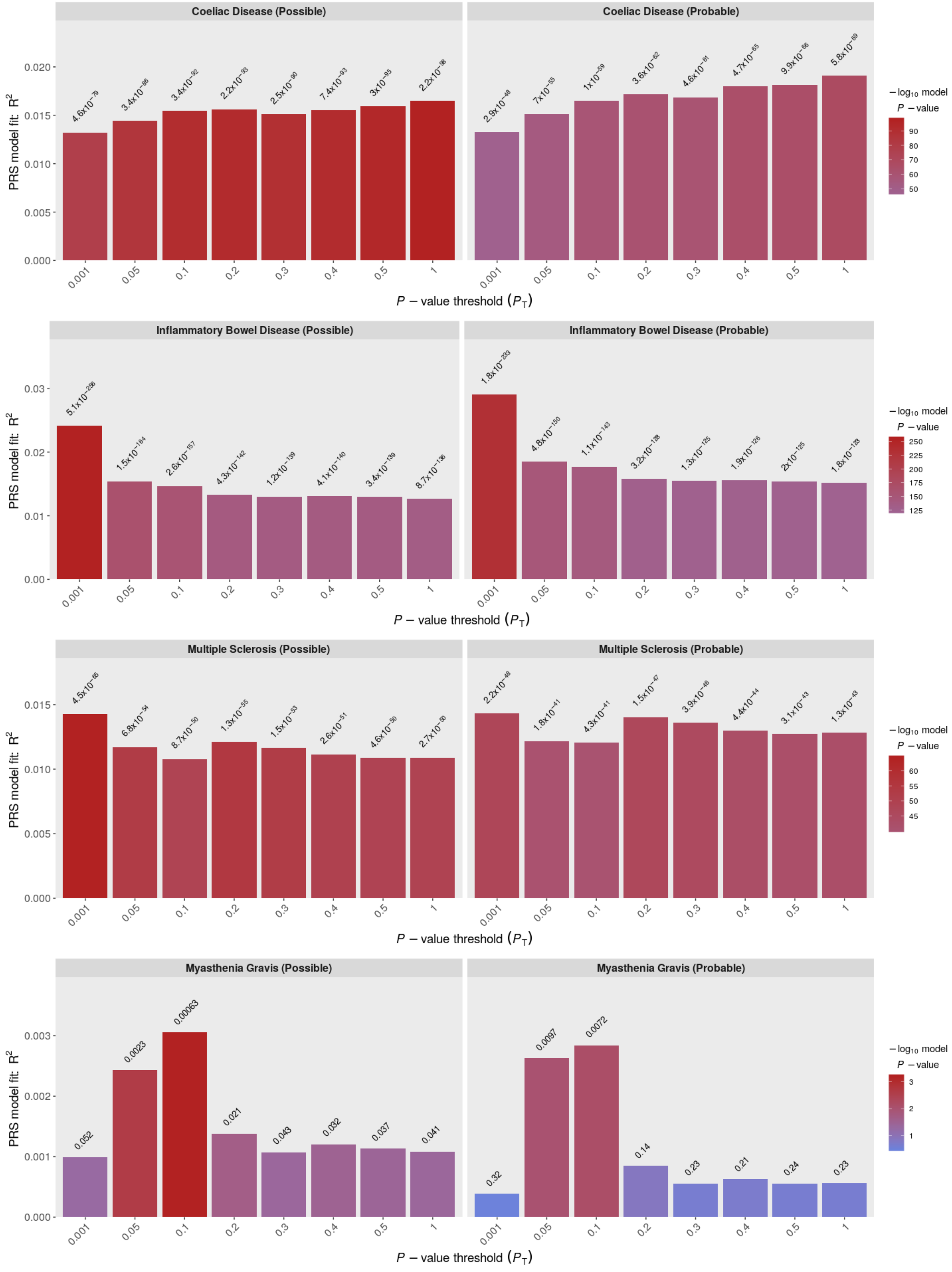
Coeliac Disease (Possible)
Coeliac Disease (Probable)
$4.6x10^{-79}$ $3.4x10^{-86}$ $3.4x10^{-92}$ $2.2x10^{-93}$ $2.5x10^{-90}$ $7.4x10^{-93}$ $3x10^{-95}$ $2.2x10^{-98}$
$2.9x10^{-48}$ $7x10^{-55}$ $1x10^{-59}$ $3.6x10^{-62}$ $4.6x10^{-61}$ $4.7x10^{-65}$ $9.9x10^{-66}$ $5.8x10^{-69}$
PRS model fit: R^2
P – value threshold (P_T)
$-log_{10}$ model P – value
Inflammatory Bowel Disease (Possible)
Inflammatory Bowel Disease (Probable)
$5.1x10^{-256}$ $1.5x10^{-164}$ $2.6x10^{-157}$ $4.3x10^{-142}$ $1.2x10^{-139}$ $4.1x10^{-140}$ $3.4x10^{-139}$ $8.7x10^{-136}$
$1.8x10^{-233}$ $4.8x10^{-150}$ $1.1x10^{-143}$ $3.2x10^{-128}$ $1.3x10^{-125}$ $1.9x10^{-126}$ $2x10^{-125}$ $1.8x10^{-123}$
Multiple Sclerosis (Possible)
Multiple Sclerosis (Probable)
$4.5x10^{-65}$ $6.8x10^{-54}$ $8.7x10^{-50}$ $1.3x10^{-55}$ $1.5x10^{-53}$ $2.6x10^{-51}$ $4.6x10^{-50}$ $2.7x10^{-50}$
$2.2x10^{-48}$ $1.8x10^{-41}$ $4.3x10^{-41}$ $1.5x10^{-47}$ $3.9x10^{-46}$ $4.4x10^{-44}$ $3.1x10^{-43}$ $1.3x10^{-43}$
Myasthenia Gravis (Possible)
Myasthenia Gravis (Probable)
0.052 0.0023 0.00063 0.021 0.043 0.032 0.037 0.041
0.32 0.0097 0.0072 0.14 0.23 0.21 0.24 0.23
0.001 0.05 0.1 0.2 0.3 0.4 0.5 1

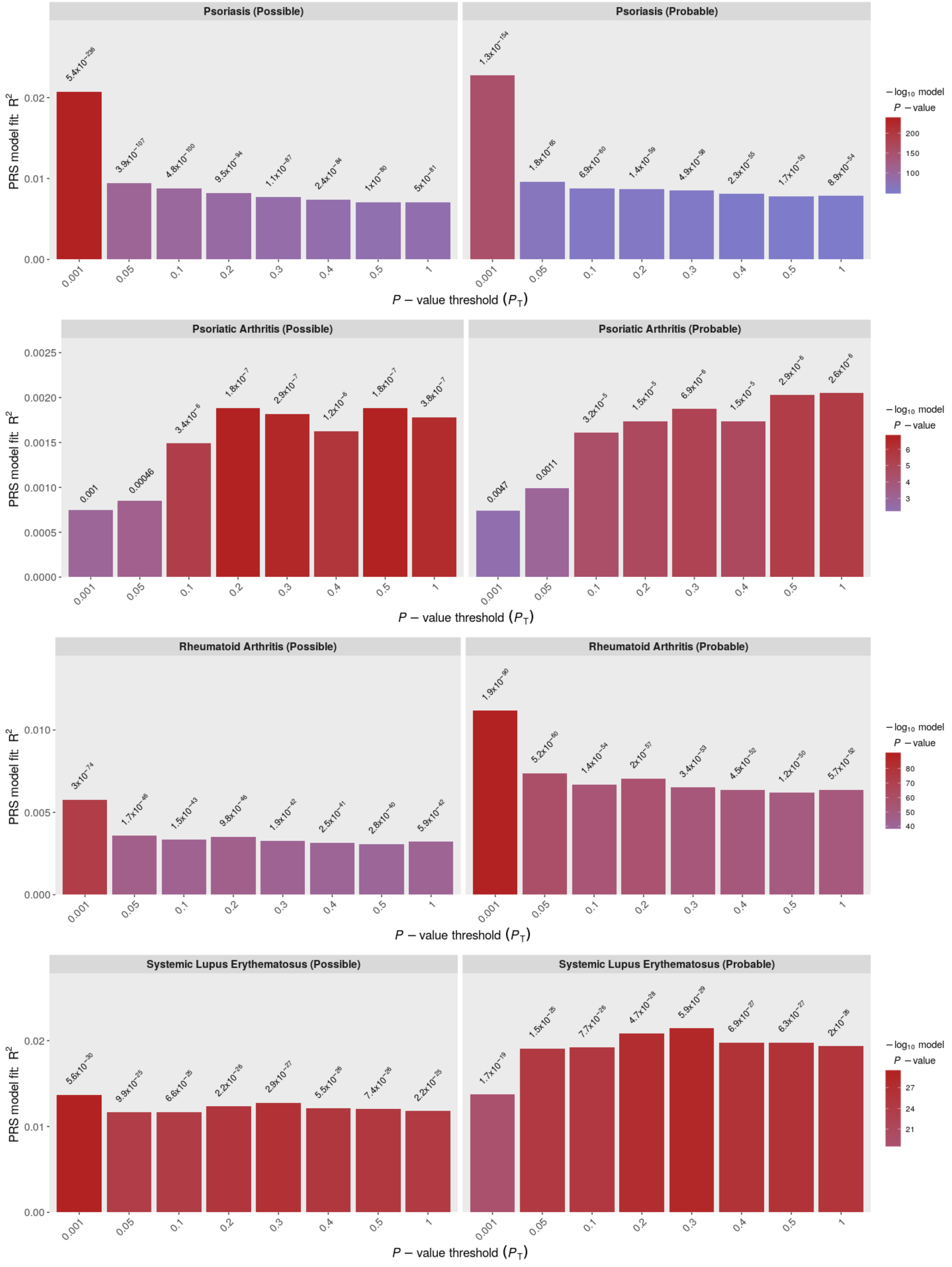

Supplementary Figure S4: Within-trait PRS - associations between PRS for autoimmune diseases and autoimmune case/control status (possible/probable) across eight P_T. Y-axis shows unadjusted R^2 (observed-scale).

Supplementary Table S4: Within-trait PRS - associations between PRS for MDD and depression case/control status (any/stringent) at optimal P_T.

Base GWAS	Target Trait in UKB	Optimal p-value threshold (P_T)	No. SNPs used to construct PRS at (P_T)	Population Prevalence used to convert to liability R^2	Unadjusted Variance explained (Observed R^2)	Adjusted Variance explained (Liability R^2)	Coefficient	Standard Error	P-value
Major Depressive Disorder	Depression (Any)	0.4	59,001	15%	1.30%	1.48%	0.23	0.005	<5e-324
Major Depressive Disorder	Depression (Stringent)	0.4	59,001	15%	1.16%	2.23%	0.28	0.009	2e-228

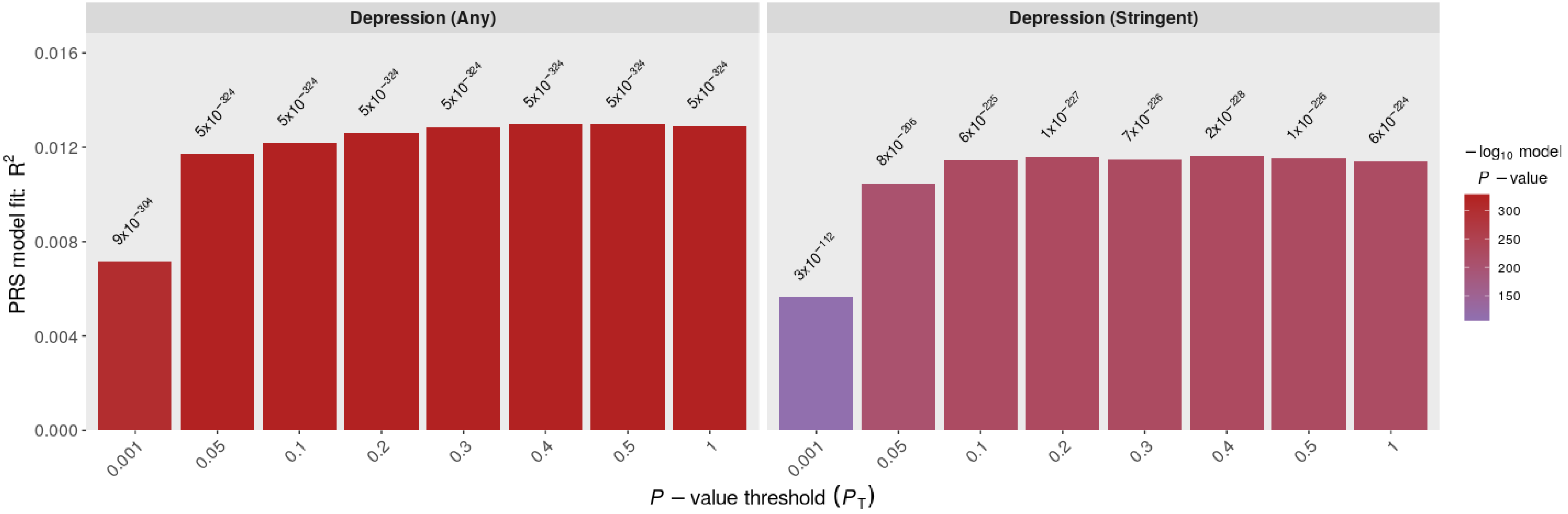

Supplementary Figure S5: Within-trait PRS - associations between PRS for MDD and depression case/control status (any/stringent) across eight P_T. Y-axis shows unadjusted R^2 (observed-scale). Note: p-value for MDD PRS predicting Depression (Any) = 0 for thresholds 0.05 to 1 due to limit on the smallest number on the R platform (5e-324).

Supplementary Table S5: Cross-trait PRS - associations between PRS for autoimmune diseases and depression case/control status (any/stringent) in men and women at optimal P_T. Associations with p-values < 7.1x10-5, meeting Bonferroni correction, highlighted in red.

Base GWAS	Target Trait in UKB	Optimal p-value threshold (P_T)	No. SNPs used to construct PRS at (P_T)	Population Prevalence used to convert to liability R^2	Unadjusted Variance explained (Observed R^2)	Adjusted Variance explained (Liability R^2)	Coefficient	Standard Error	P-value
Coeliac	Any (combined)	0.200	12,430	15%	0.0021%	0.0024%	0.009	0.005	4.4e-02
Coeliac	Any (female)	0.200	12,430	15%	0.0006%	0.0007%	0.005	0.006	4.1e-01
Coeliac	Any (male)	0.050	3,881	15%	0.0045%	0.0060%	0.015	0.008	5.4e-02
Coeliac	Stringent (combined)	0.200	12,430	15%	0.0013%	0.0026%	0.010	0.009	2.7e-01
Coeliac	Stringent (female)	0.001	186	15%	0.0017%	0.0029%	0.010	0.011	3.4e-01
Coeliac	Stringent (male)	0.001	186	15%	0.0034%	0.0075%	-0.016	0.015	2.7e-01
Inflammatory Bowel Disease	Any (combined)	1.000	80,201	15%	0.0051%	0.0058%	0.014	0.005	1.6e-03
Inflammatory Bowel Disease	Any (female)	1.000	80,201	15%	0.0047%	0.0049%	0.013	0.006	2.4e-02
Inflammatory Bowel Disease	Any (male)	1.000	80,201	15%	0.0079%	0.0106%	0.019	0.008	1.0e-02
Inflammatory Bowel Disease	Stringent (combined)	0.300	38,677	15%	0.0173%	0.0333%	0.035	0.009	7.9e-05
Inflammatory Bowel Disease	Stringent (female)	0.050	10,082	15%	0.0238%	0.0406%	0.038	0.011	4.4e-04
Inflammatory Bowel Disease	Stringent (male)	1.000	80,201	15%	0.0230%	0.0510%	0.043	0.015	4.2e-03
Lupus	Any (combined)	0.001	836	15%	0.0029%	0.0034%	-0.011	0.004	1.7e-02
Lupus	Any (female)	0.001	836	15%	0.0026%	0.0027%	-0.010	0.006	9.4e-02
Lupus	Any (male)	0.001	836	15%	0.0056%	0.0075%	-0.016	0.007	3.1e-02
Lupus	Stringent (combined)	0.001	836	15%	0.0018%	0.0035%	-0.011	0.009	2.0e-01
Lupus	Stringent (female)	0.001	836	15%	0.0038%	0.0066%	-0.015	0.011	1.6e-01
Lupus	Stringent (male)	0.050	18,572	15%	0.0020%	0.0045%	0.013	0.015	3.9e-01
Multiple Sclerosis	Any (combined)	0.050	3,240	15%	0.0052%	0.0059%	0.014	0.005	1.5e-03
Multiple Sclerosis	Any (female)	0.050	3,240	15%	0.0066%	0.0068%	0.015	0.006	7.4e-03
Multiple Sclerosis	Any (male)	0.050	3,240	15%	0.0058%	0.0078%	0.016	0.007	2.8e-02
Multiple Sclerosis	Stringent (combined)	0.050	3,240	15%	0.0033%	0.0063%	0.015	0.009	8.6e-02
Multiple Sclerosis	Stringent (female)	0.100	5,974	15%	0.0087%	0.0148%	0.023	0.011	3.4e-02
Multiple Sclerosis	Stringent (male)	0.050	3,240	15%	0.0024%	0.0053%	0.014	0.015	3.6e-01
Myasthenia Gravis	Any (combined)	0.300	56,894	15%	0.0084%	0.0096%	0.018	0.005	5.2e-05
Myasthenia Gravis	Any (female)	0.200	40,875	15%	0.0127%	0.0131%	0.022	0.006	2.1e-04
Myasthenia Gravis	Any (male)	0.300	56,894	15%	0.0073%	0.0098%	0.019	0.008	1.4e-02
Myasthenia Gravis	Stringent (combined)	0.300	56,894	15%	0.0208%	0.0399%	0.038	0.009	1.6e-05
Myasthenia Gravis	Stringent (female)	0.300	56,894	15%	0.0164%	0.0279%	0.032	0.011	3.5e-03
Myasthenia Gravis	Stringent (male)	0.300	56,894	15%	0.0384%	0.0849%	0.055	0.015	2.2e-04

Base GWAS	Target Trait in UKB	Optimal p-value threshold (P_T)	No. SNPs used to construct PRS at (P_T)	Population Prevalence used to convert to liability R^2	Unadjusted Variance explained (Observed R^2)	Adjusted Variance explained (Liability R^2)	Coefficient	Standard Error	P-value
Psoriasis	Any (combined)	1.000	136,084	15%	0.0101%	0.0116%	0.020	0.005	8.7e-06
Psoriasis	Any (female)	1.000	136,084	15%	0.0124%	0.0128%	0.021	0.006	2.5e-04
Psoriasis	Any (male)	0.050	13,899	15%	0.0133%	0.0178%	0.025	0.007	8.9e-04
Psoriasis	Stringent (combined)	0.050	13,899	15%	0.0126%	0.0243%	0.029	0.009	7.6e-04
Psoriasis	Stringent (female)	1.000	136,084	15%	0.0177%	0.0303%	0.033	0.011	2.4e-03
Psoriasis	Stringent (male)	0.050	13,899	15%	0.0093%	0.0207%	0.027	0.015	6.8e-02
Psoriatic Arthritis	Any (combined)	0.050	4,666	15%	0.0013%	0.0015%	-0.010	0.007	1.1e-01
Psoriatic Arthritis	Any (female)	1.000	45,914	15%	0.0026%	0.0027%	0.018	0.011	9.2e-02
Psoriatic Arthritis	Any (male)	0.100	8,135	15%	0.0058%	0.0077%	-0.026	0.012	2.8e-02
Psoriatic Arthritis	Stringent (combined)	0.001	211	15%	0.0018%	0.0036%	0.012	0.009	2.0e-01
Psoriatic Arthritis	Stringent (female)	1.000	45,914	15%	0.0086%	0.0147%	0.042	0.020	3.4e-02
Psoriatic Arthritis	Stringent (male)	0.100	8,135	15%	0.0081%	0.0178%	-0.039	0.023	9.1e-02
Rheumatoid Arthritis	Any (combined)	1.000	118,649	15%	0.0006%	0.0007%	0.005	0.005	2.6e-01
Rheumatoid Arthritis	Any (female)	0.001	550	15%	0.0011%	0.0011%	-0.006	0.006	2.8e-01
Rheumatoid Arthritis	Any (male)	1.000	118,649	15%	0.0059%	0.0079%	0.017	0.008	2.7e-02
Rheumatoid Arthritis	Stringent (combined)	1.000	118,649	15%	0.0095%	0.0183%	0.025	0.009	3.4e-03
Rheumatoid Arthritis	Stringent (female)	0.001	550	15%	0.0047%	0.0080%	0.017	0.011	1.2e-01
Rheumatoid Arthritis	Stringent (male)	1.000	118,649	15%	0.0232%	0.0514%	0.043	0.015	4.1e-03

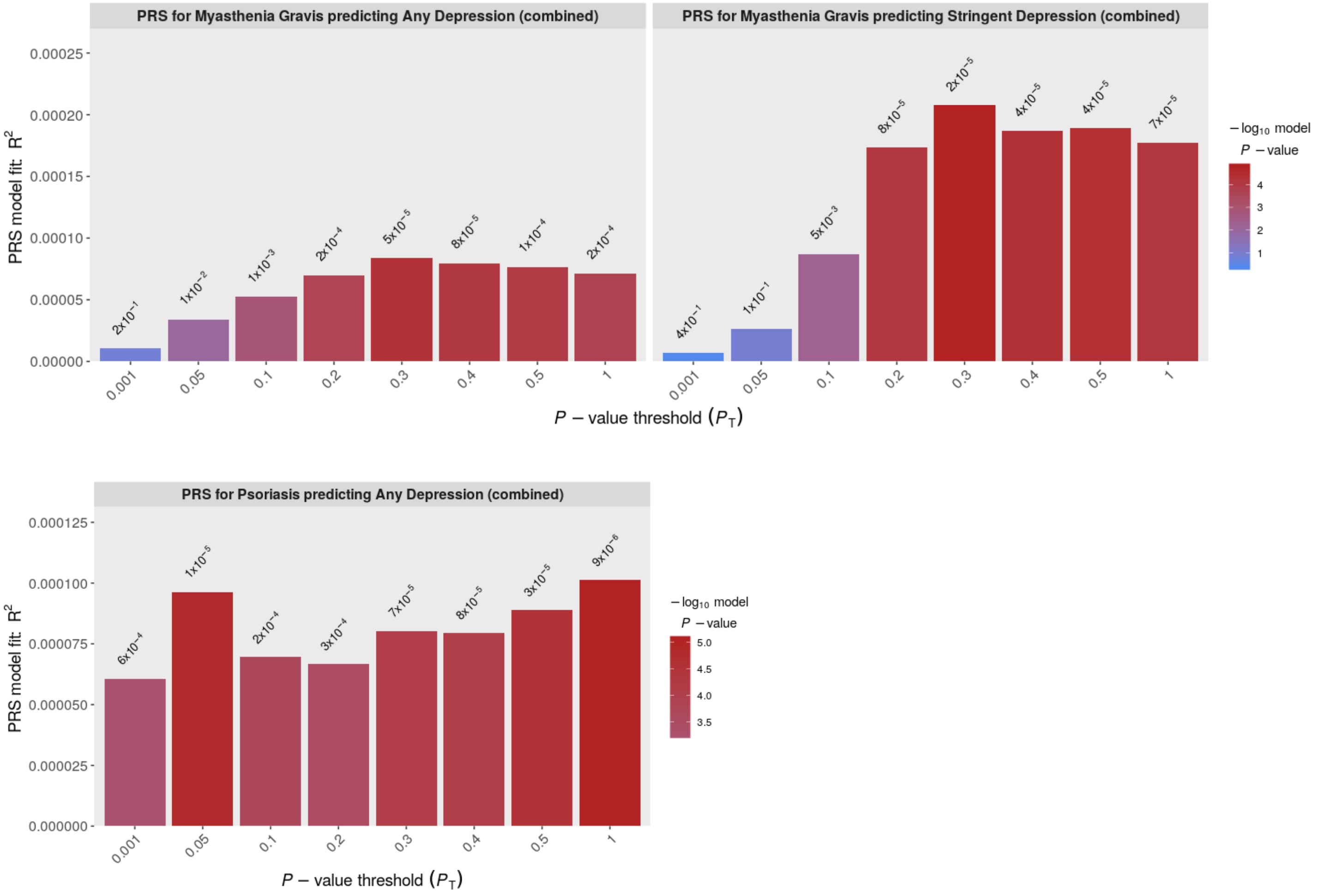

Supplementary Figure S6: Cross-trait PRS –associations between PRS for autoimmune diseases and depression case/control status (any/stringent) in men and women across eight P_T. Y-axis shows unadjusted R^2 (observed-scale). Bar charts are shown for significant results only.

Supplementary Table S6: Cross-trait PRS - associations between PRS for MDD and autoimmune case/control status (possible/probable) in men and women at optimal P_T. MDD = Major Depressive Disorder. Associations with p-values < 7.1x10^{-5}, meeting Bonferroni correction, highlighted in red. Sex*PRS p-value – estimated where significant associations were observed in sex-specific analyses; tested for an interaction between sex and PRS in the full sample (Phenotype ~ sex + PRS + sex*PRS + covariates).

Base GWAS	Target Trait in UKB	Optimal p-value threshold (P_T)	No. SNPs used to construct PRS at (P_T)	Population Prevalence used to convert to liability R^2	Unadjusted Variance explained (Observed R^2)	Adjusted Variance explained (Liability R^2)	Coefficient	Standard Error	P-value	Sex*PRS p-value
MDD	Ankylosing Spondylitis, Possible (combined)	0.050	14,137	0.55%	0.035%	0.054%	0.067	0.027	1.4e-02	NA
MDD	Ankylosing Spondylitis, Possible (female)	0.001	1,123	0.55%	0.130%	0.210%	0.132	0.044	2.8e-03	NA
MDD	Ankylosing Spondylitis, Possible (male)	1.000	99,216	0.55%	0.043%	0.063%	0.072	0.035	3.9e-02	NA
MDD	Ankylosing Spondylitis, Probable (combined)	0.001	1,123	0.55%	0.003%	0.006%	-0.022	0.049	6.5e-01	NA
MDD	Ankylosing Spondylitis, Probable (female)	0.400	59,001	0.55%	0.032%	0.064%	-0.074	0.096	4.5e-01	NA
MDD	Ankylosing Spondylitis, Probable (male)	0.001	1,123	0.55%	0.005%	0.008%	-0.026	0.057	6.5e-01	NA
MDD	Autoimmune Thyroid Disease, Possible (combined)	0.500	67,956	2.00%	0.078%	0.173%	0.104	0.034	2.6e-03	NA
MDD	Autoimmune Thyroid Disease, Possible (female)	0.500	67,956	2.00%	0.075%	0.154%	0.098	0.037	8.8e-03	NA
MDD	Autoimmune Thyroid Disease, Possible (male)	0.500	67,956	2.00%	0.110%	0.278%	0.133	0.087	1.3e-01	NA
MDD	Autoimmune Thyroid Disease, Probable (combined)	0.400	59,001	2.00%	0.083%	0.193%	0.110	0.041	7.3e-03	NA
MDD	Autoimmune Thyroid Disease, Probable (female)	0.400	59,001	2.00%	0.082%	0.178%	0.105	0.044	1.7e-02	NA
MDD	Autoimmune Thyroid Disease, Probable (male)	0.001	1,123	2.00%	0.138%	0.363%	-0.153	0.108	1.5e-01	NA
MDD	Coeliac, Possible (combined)	0.001	1,123	1.00%	0.109%	0.172%	-0.112	0.021	6.0e-08	NA
MDD	Coeliac, Possible (female)	0.001	1,123	1.00%	0.147%	0.221%	-0.127	0.025	5.4e-07	0.33
MDD	Coeliac, Possible (male)	0.001	1,123	1.00%	0.054%	0.091%	-0.082	0.036	2.3e-02	NA
MDD	Coeliac, Probable (combined)	0.001	1,123	1.00%	0.059%	0.104%	-0.087	0.028	2.0e-03	NA
MDD	Coeliac, Probable (female)	0.001	1,123	1.00%	0.066%	0.110%	-0.090	0.034	8.7e-03	NA
MDD	Coeliac, Probable (male)	0.001	1,123	1.00%	0.048%	0.090%	-0.081	0.050	1.1e-01	NA

Base GWAS	Target Trait in UKB	Optimal p-value threshold (P_T)	No. SNPs used to construct PRS at (P_T)	Population Prevalence used to convert to liability R^2	Unadjusted Variance explained (Observed R^2)	Adjusted Variance explained (Liability R^2)	Coefficient	Standard Error	P-value	Sex*PRS p-value
MDD	Inflammatory Bowel Disease, Possible (combined)	0.300	48,852	0.50%	0.051%	0.058%	0.071	0.014	6.5e-07	NA
MDD	Inflammatory Bowel Disease, Possible (female)	0.050	14,137	0.50%	0.103%	0.119%	0.100	0.020	4.0e-07	0.02
MDD	Inflammatory Bowel Disease, Possible (male)	0.300	48,852	0.50%	0.044%	0.051%	0.066	0.020	1.2e-03	NA
MDD	Inflammatory Bowel Disease, Probable (combined)	0.050	14,137	0.50%	0.029%	0.036%	0.055	0.017	1.2e-03	NA
MDD	Inflammatory Bowel Disease, Probable (female)	0.050	14,137	0.50%	0.083%	0.104%	0.094	0.024	9.0e-05	NA
MDD	Inflammatory Bowel Disease, Probable (male)	1.000	99,216	0.50%	0.014%	0.017%	0.038	0.024	1.1e-01	NA
MDD	Multiple Sclerosis, Possible (combined)	0.500	67,956	0.10%	0.047%	0.051%	0.076	0.025	1.9e-03	NA
MDD	Multiple Sclerosis, Possible (female)	0.001	1,123	0.10%	0.058%	0.059%	0.082	0.029	4.4e-03	NA
MDD	Multiple Sclerosis, Possible (male)	0.050	14,137	0.10%	0.115%	0.136%	0.125	0.047	8.1e-03	NA
MDD	Multiple Sclerosis, Probable (combined)	1.000	99,216	0.10%	0.064%	0.073%	0.091	0.030	2.0e-03	NA
MDD	Multiple Sclerosis, Probable (female)	0.001	1,123	0.10%	0.081%	0.088%	0.100	0.035	3.9e-03	NA
MDD	Multiple Sclerosis, Probable (male)	0.050	14,137	0.10%	0.160%	0.201%	0.152	0.057	7.6e-03	NA
MDD	Myasthenia Gravis, Possible (combined)	1.000	99,216	0.02%	0.045%	0.052%	0.087	0.066	1.9e-01	NA
MDD	Myasthenia Gravis, Possible (female)	1.000	99,216	0.02%	0.178%	0.202%	0.171	0.088	5.1e-02	NA
MDD	Myasthenia Gravis, Possible (male)	0.100	22,831	0.02%	0.014%	0.016%	-0.048	0.099	6.3e-01	NA
MDD	Myasthenia Gravis, Probable (combined)	0.001	1,123	0.02%	0.014%	0.017%	0.050	0.082	5.5e-01	NA
MDD	Myasthenia Gravis, Probable (female)	1.000	99,216	0.02%	0.075%	0.092%	0.115	0.119	3.3e-01	NA
MDD	Myasthenia Gravis, Probable (male)	1.000	99,216	0.02%	0.071%	0.085%	-0.112	0.116	3.4e-01	NA
MDD	Pernicious Anemia, Possible (combined)	0.050	14,137	0.10%	0.035%	0.038%	0.066	0.025	9.4e-03	NA

Base GWAS	Target Trait in UKB	Optimal p-value threshold (P_T)	No. SNPs used to construct PRS at (P_T)	Population Prevalence used to convert to liability R^2	Unadjusted Variance explained (Observed R^2)	Adjusted Variance explained (Liability R^2)	Coefficient	Standard Error	P-value	Sex*PRS p-value
MDD	Pernicious Anemia, Possible (female)	0.200	36,975	0.10%	0.030%	0.031%	0.059	0.030	5.0e-02	NA
MDD	Pernicious Anemia, Possible (male)	0.500	67,956	0.10%	0.059%	0.070%	0.090	0.048	6.0e-02	NA
MDD	Pernicious Anemia, Probable (combined)	0.050	14,137	0.10%	0.113%	0.150%	0.131	0.049	7.2e-03	NA
MDD	Pernicious Anemia, Probable (female)	0.100	22,831	0.10%	0.064%	0.082%	0.097	0.057	9.3e-02	NA
MDD	Pernicious Anemia, Probable (male)	0.050	14,137	0.10%	0.380%	0.544%	0.250	0.093	6.9e-03	NA
MDD	Polymyalgia Rheumatica/GCA, Possible (combined)	0.200	36,975	0.85%	0.058%	0.095%	0.085	0.025	6.8e-04	NA
MDD	Polymyalgia Rheumatica/GCA, Possible (female)	0.200	36,975	0.85%	0.114%	0.177%	0.116	0.031	1.5e-04	NA
MDD	Polymyalgia Rheumatica/GCA, Possible (male)	0.001	1,123	0.85%	0.007%	0.012%	0.030	0.043	4.8e-01	NA
MDD	Polymyalgia Rheumatica/GCA, Probable (combined)	0.200	36,975	0.85%	0.074%	0.132%	0.100	0.034	2.8e-03	NA
MDD	Polymyalgia Rheumatica/GCA, Probable (female)	0.200	36,975	0.85%	0.110%	0.189%	0.120	0.041	3.3e-03	NA
MDD	Polymyalgia Rheumatica/GCA, Probable (male)	0.001	1,123	0.85%	0.113%	0.214%	0.128	0.059	3.0e-02	NA
MDD	Psoriasis, Possible (combined)	0.500	67,956	2.00%	0.061%	0.095%	0.076	0.014	2.5e-08	NA
MDD	Psoriasis, Possible (female)	0.500	67,956	2.00%	0.062%	0.100%	0.079	0.020	1.0e-04	NA
MDD	Psoriasis, Possible (male)	0.500	67,956	2.00%	0.060%	0.091%	0.075	0.019	5.8e-05	0.86
MDD	Psoriasis, Probable (combined)	0.500	67,956	2.00%	0.077%	0.139%	0.093	0.019	1.4e-06	NA
MDD	Psoriasis, Probable (female)	0.500	67,956	2.00%	0.118%	0.223%	0.117	0.030	7.9e-05	NA
MDD	Psoriasis, Probable (male)	0.050	14,137	2.00%	0.061%	0.106%	0.081	0.025	1.3e-03	NA
MDD	Psoriatic Arthritis, Possible (combined)	1.000	99,216	0.50%	0.105%	0.163%	0.118	0.030	9.7e-05	NA
MDD	Psoriatic Arthritis, Possible (female)	1.000	99,216	0.50%	0.159%	0.248%	0.145	0.043	6.5e-04	NA
MDD	Psoriatic Arthritis, Possible (male)	0.001	1,123	0.50%	0.069%	0.107%	0.095	0.043	2.6e-02	NA
MDD	Psoriatic Arthritis, Probable (combined)	0.300	48,852	0.50%	0.167%	0.274%	0.153	0.036	2.2e-05	NA

Base GWAS	Target Trait in UKB	Optimal p-value threshold (P_T)	No. SNPs used to construct PRS at (P_T)	Population Prevalence used to convert to liability R^2	Unadjusted Variance explained (Observed R^2)	Adjusted Variance explained (Liability R^2)	Coefficient	Standard Error	P-value	Sex*PRS p-value
MDD	Psoriatic Arthritis, Probable (female)	1.000	99,216	0.50%	0.212%	0.349%	0.173	0.051	6.4e-04	NA
MDD	Psoriatic Arthritis, Probable (male)	0.001	1,123	0.50%	0.153%	0.250%	0.146	0.051	4.4e-03	NA
MDD	Rheumatoid Arthritis, Possible (combined)	0.500	67,956	1.00%	0.065%	0.082%	0.078	0.013	1.1e-09	NA
MDD	Rheumatoid Arthritis, Possible (female)	0.300	48,852	1.00%	0.060%	0.071%	0.072	0.016	3.5e-06	0.68
MDD	Rheumatoid Arthritis, Possible (male)	1.000	99,216	1.00%	0.090%	0.125%	0.096	0.022	1.6e-05	0.26
MDD	Rheumatoid Arthritis, Probable (combined)	0.100	22,831	1.00%	0.065%	0.095%	0.083	0.017	1.2e-06	NA
MDD	Rheumatoid Arthritis, Probable (female)	0.300	48,852	1.00%	0.070%	0.096%	0.084	0.021	4.5e-05	0.90
MDD	Rheumatoid Arthritis, Probable (male)	1.000	99,216	1.00%	0.075%	0.121%	0.094	0.032	2.9e-03	NA
MDD	Sjögren Syndrome, Possible (combined)	0.200	36,975	0.10%	0.064%	0.080%	0.096	0.040	1.5e-02	NA
MDD	Sjögren Syndrome, Possible (female)	0.200	36,975	0.10%	0.041%	0.047%	0.073	0.042	7.9e-02	NA
MDD	Sjögren Syndrome, Possible (male)	0.050	14,137	0.10%	0.517%	0.799%	0.305	0.128	1.8e-02	NA
MDD	Sjögren Syndrome, Probable (combined)	1.000	99,216	0.10%	0.042%	0.057%	0.081	0.051	1.1e-01	NA
MDD	Sjögren Syndrome, Probable (female)	0.200	36,975	0.10%	0.028%	0.035%	0.064	0.054	2.4e-01	NA
MDD	Sjögren Syndrome, Probable (male)	0.500	67,956	0.10%	0.375%	0.605%	0.265	0.158	9.3e-02	NA
MDD	Systemic Lupus Erythematosus, Possible (combined)	0.050	14,137	0.10%	0.097%	0.123%	0.119	0.040	3.2e-03	NA
MDD	Systemic Lupus Erythematosus, Possible (female)	0.050	14,137	0.10%	0.111%	0.130%	0.122	0.044	5.3e-03	NA
MDD	Systemic Lupus Erythematosus, Possible (male)	1.000	99,216	0.10%	0.180%	0.265%	0.175	0.102	8.7e-02	NA
MDD	Systemic Lupus Erythematosus, Probable (combined)	0.050	14,137	0.10%	0.170%	0.231%	0.163	0.053	2.1e-03	NA

Base GWAS	Target Trait in UKB	Optimal p-value threshold (P_T)	No. SNPs used to construct PRS at (P_T)	Population Prevalence used to convert to liability R^2	Unadjusted Variance explained (Observed R^2)	Adjusted Variance explained (Liability R^2)	Coefficient	Standard Error	P-value	Sex*PRS p-value
MDD	Systemic Lupus Erythematosus, Probable (female)	0.050	14,137	0.10%	0.190%	0.242%	0.166	0.057	3.5e-03	NA
MDD	Systemic Lupus Erythematosus, Probable (male)	0.500	67,956	0.10%	0.154%	0.243%	0.168	0.141	2.3e-01	NA
MDD	Type 1 Diabetes, Possible (combined)	0.400	59,001	0.30%	0.082%	0.098%	0.096	0.019	6.0e-07	NA
MDD	Type 1 Diabetes, Possible (female)	1.000	99,216	0.30%	0.126%	0.156%	0.121	0.030	4.5e-05	0.18
MDD	Type 1 Diabetes, Possible (male)	0.300	48,852	0.30%	0.066%	0.075%	0.084	0.025	8.7e-04	NA
MDD	Type 1 Diabetes, Probable (combined)	0.500	67,956	0.30%	0.089%	0.110%	0.102	0.021	1.4e-06	NA
MDD	Type 1 Diabetes, Probable (female)	1.000	99,216	0.30%	0.117%	0.149%	0.119	0.032	2.2e-04	NA
MDD	Type 1 Diabetes, Probable (male)	0.300	48,852	0.30%	0.081%	0.097%	0.095	0.028	6.1e-04	NA

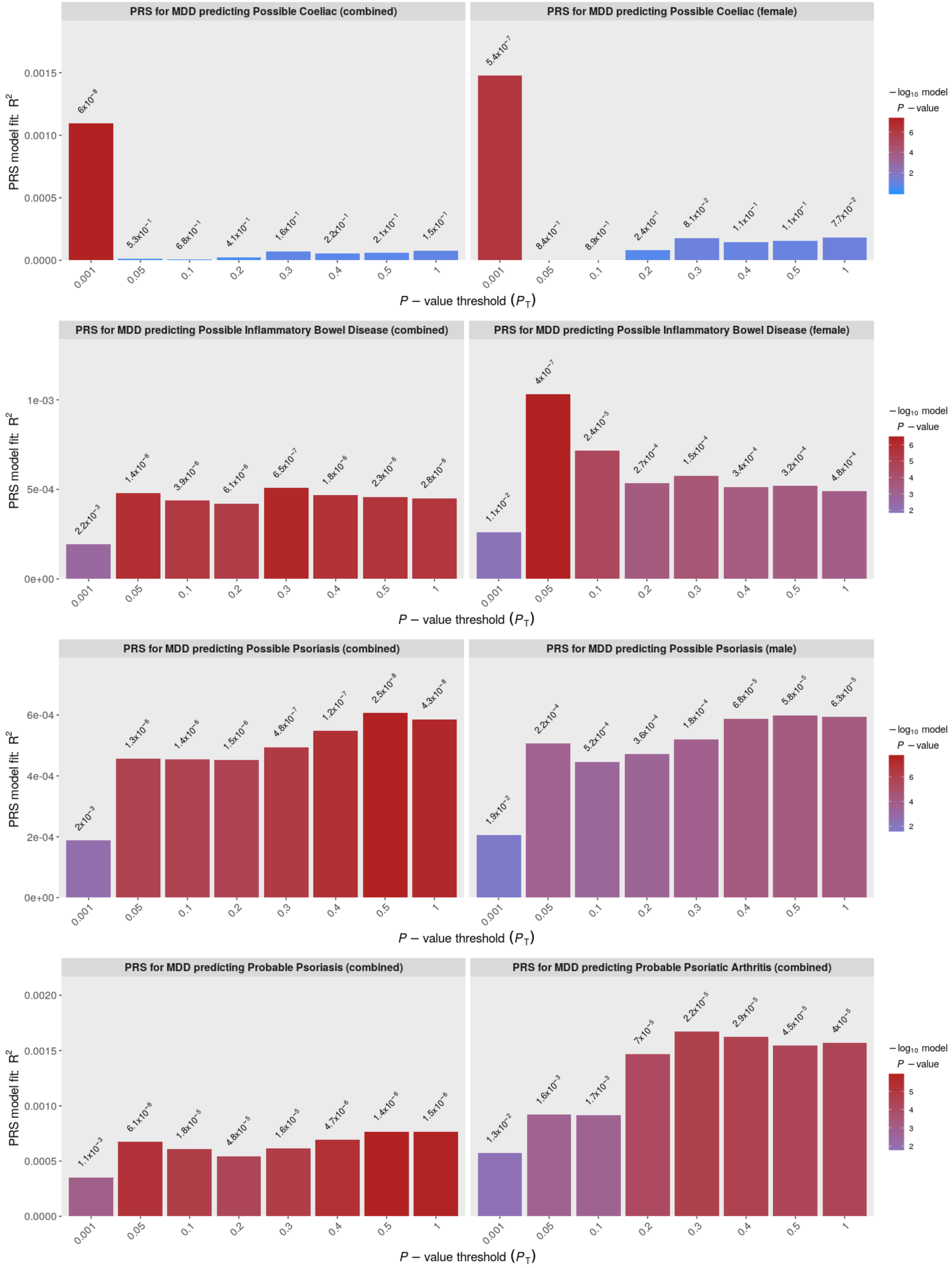
PRS for MDD predicting Possible Coeliac (combined)
PRS for MDD predicting Possible Coeliac (female)
PRS model fit: R^2
$-\log_{10}$ model P − value
6x10^-8
5.3x10^-1
6.8x10^-1
4.1x10^-1
1.6x10^-1
2.2x10^-1
2.1x10^-1
1.5x10^-1
5.4x10^-7
8.4x10^-1
8.9x10^-1
2.4x10^-1
8.1x10^-2
1.1x10^-1
1.1x10^-1
7.7x10^-2
P − value threshold (P_T)
PRS for MDD predicting Possible Inflammatory Bowel Disease (combined)
PRS for MDD predicting Possible Inflammatory Bowel Disease (female)
2.2x10^-3
1.4x10^-6
3.9x10^-6
6.1x10^-6
6.5x10^-7
1.8x10^-6
2.3x10^-6
2.8x10^-6
1.1x10^-2
4x10^-7
2.4x10^-5
2.7x10^-4
1.5x10^-4
3.4x10^-4
3.2x10^-4
4.8x10^-4
PRS for MDD predicting Possible Psoriasis (combined)
PRS for MDD predicting Possible Psoriasis (male)
2x10^-3
1.3x10^-6
1.4x10^-6
1.5x10^-6
4.8x10^-7
1.2x10^-7
2.5x10^-8
4.3x10^-8
1.9x10^-2
2.2x10^-4
5.2x10^-4
3.6x10^-4
1.8x10^-4
6.8x10^-5
5.8x10^-5
6.3x10^-5
PRS for MDD predicting Probable Psoriasis (combined)
PRS for MDD predicting Probable Psoriatic Arthritis (combined)
1.1x10^-3
6.1x10^-6
1.8x10^-5
4.8x10^-5
1.6x10^-5
4.7x10^-6
1.4x10^-6
1.5x10^-6
1.3x10^-2
1.6x10^-3
1.7x10^-3
7x10^-5
2.2x10^-5
2.9x10^-5
4.5x10^-5
4x10^-5
0.001 0.05 0.1 0.2 0.3 0.4 0.5 1

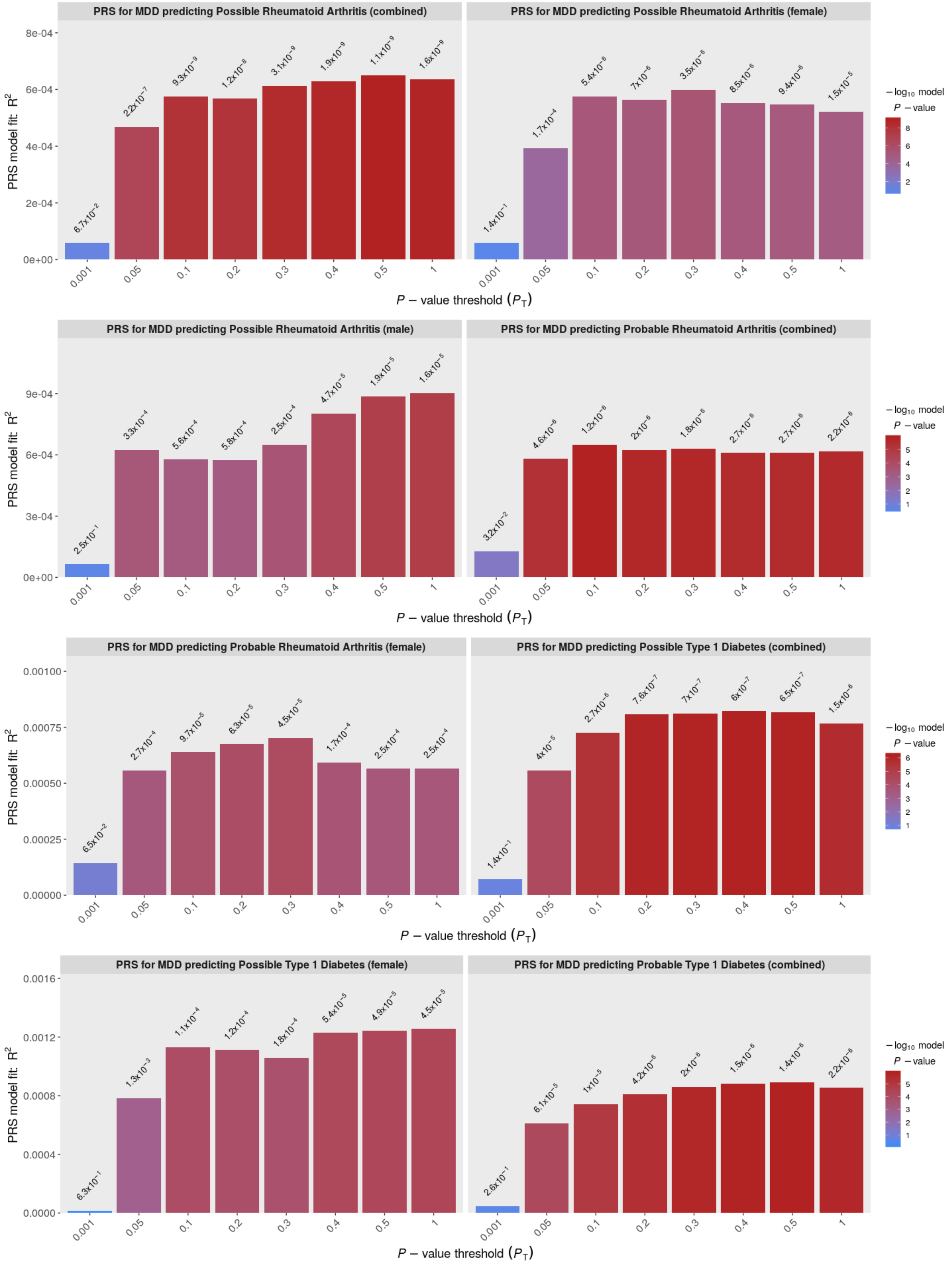

Supplementary Figure S7: Cross-trait PRS –associations between PRS for MDD and autoimmune case/control status (possible/probable) in men and women across eight P_T. Y-axis shows unadjusted R^2 (observed-scale). Bar charts are shown for significant results only.

3 Genetic correlations

Supplementary Table S7: Genetic correlations between autoimmune traits and ‘any’ and ‘stringent’ depression in the UKB. Associations with p-values < 4.1×10^{-3}, meeting Bonferroni correction, highlighted in red.

	r_G	95% CIs	P-value	r_G	95% CIs	P-value
Autoimmune Disease	Any depression			Stringent depression		
Coeliac Disease	0.017	-0.10 - 0.14	7.8e-01	0.048	-0.10 - 0.19	5.2e-01
Inflammatory Bowel Disease	0.106	0.03 - 0.18	3.8e-03	0.157	0.07 - 0.24	3.0e-04
Multiple Sclerosis	0.302	0.06 - 0.55	1.6e-02	0.375	0.07 - 0.68	1.5e-02
Psoriasis	0.064	-0.01 - 0.14	9.0e-02	0.161	0.06 - 0.26	1.1e-03
Rheumatoid Arthritis	0.004	-0.06 - 0.07	9.0e-01	0.104	0.02 - 0.19	1.7e-02
Systemic Lupus Erythematosus	-0.001	-0.11 - 0.10	9.9e-01	0.126	0.00 - 0.25	5.1e-02
