# Supplementary material for: Investigating Pleiotropy Between Depression and Autoimmune Diseases Using the UK Biobank
Source: Biol Psychiatry Glob Open Sci. 2021 Mar 25;1(1):48–58. doi: 10.1016/j.bpsgos.2021.03.002 (PMC8262258; doi:10.1016/j.bpsgos.2021.03.002)
Supplement: Supplement 2 [file mmc2.zip › bpsgos_4_Supplement 2_Glanville et al_mmc2.html]

Investigating pleiotropy between depression and autoimmune diseases using the UK Biobank, applying strict and minimal phenotyping, Supplementary Material


# Investigating pleiotropy between depression and autoimmune diseases using the UK Biobank, applying strict and minimal phenotyping, Supplementary Material

# 1 Definition of each autoimmune disease

## 1.1 Pernicious Anemia

---

**Venn diagrams:** *Left:* probable cases as a subset of possible; *Right:* overlapping measures in the UK Biobank

**Matrix of ICD-10 primary and secondary dx - cells show number of individuals with each combination of dx**

|  | 0 primary dx | 1 primary dx | >1 primary dx |
| --- | --- | --- | --- |
| 0 secondary dx | 324,0741 | 242 | 33 |
| 1 secondary dx | 4272 | 33 | 13 |
| >1 secondary dx | 2193 | 33 | 13 |
|  |
| --- |
| Note: |
|  |
| 1 Controls |
| 2 Possible cases |
| 3 Probable cases |

**ICD-10 codes used to construct matrix above. Columns show count of individuals who have received at least one dx**

| ICD-10 sub-codes | Primary dx | Secondary dx |
| --- | --- | --- |
| **D51 Vitamin B 12 deficiency anaemia** | | |
| D51.0 Vitamin B12 deficiency anaemia due to intrinsic factor deficiency | 35 | 654 |

**Count of self-reported autoimmune condition**

| Self-reported disorder | Number of people |
| --- | --- |
| Pernicious anaemia | 1,209 |

## 1.2 Autoimmune Thyroid Disease

---

**Venn diagrams:** *Left:* probable cases as a subset of possible; *Right:* overlapping measures in the UK Biobank

**Matrix of ICD-10 primary and secondary dx - cells show number of individuals with each combination of dx**

|  | 0 primary dx | 1 primary dx | >1 primary dx |
| --- | --- | --- | --- |
| 0 secondary dx | 324,0741 | 1472 | 203 |
| 1 secondary dx | 2832 | 253 | 53 |
| >1 secondary dx | 1033 | 113 | 193 |
|  |
| --- |
| Note: |
|  |
| 1 Controls |
| 2 Possible cases |
| 3 Probable cases |

**ICD-10 codes used to construct matrix above. Columns show count of individuals who have received at least one dx**

| ICD-10 sub-codes | Primary dx | Secondary dx |
| --- | --- | --- |
| **E05 Thyrotoxicosis [hyperthyroidism]** | | |
| E05.0 Thyrotoxicosis with diffuse goitre | 169 | 297 |
| **E06 Thyroiditis** | | |
| E06.3 Autoimmune thyroiditis | 58 | 150 |

**Count of self-reported autoimmune condition**

| Self-reported disorder | Number of people |
| --- | --- |
| Thyroiditis | 200 |
| Grave’s disease | 100 |

**Count of self-reported prescription medications**

| Disease | Medication | Count |
| --- | --- | --- |
| Autoimmune Thyroid Disease (Grave’s and Thyroiditis) | levothyroxine sodium | 16,280 |
|  | thyroxine product | 4,605 |
|  | thyroxine sodium | 1,102 |
|  | carbimazole | 324 |
|  | sodium thyroxine | 183 |
|  | liothyronine | 95 |
|  | eltroxin 25micrograms tablet | 90 |
|  | propylthiouracil | 13 |
|  | sodium liothyronine | 9 |
|  | t3 - liothyronine | 6 |
|  | propylthiouracil product | 6 |
|  | neo-mercazole 5mg tablet | 4 |
|  | tertroxin 20mcg tablet | 4 |
|  | protirelin | 1 |

## 1.3 Type 1 diabetes

---

**Venn diagrams:** *Left:* probable cases as a subset of possible; *Right:* overlapping measures in the UK Biobank

**Matrix of ICD-10 primary and secondary dx - cells show number of individuals with each combination of dx**

|  | 0 primary dx | 1 primary dx | >1 primary dx |
| --- | --- | --- | --- |
| 0 secondary dx | 324,0741 | 1192 | 373 |
| 1 secondary dx | 1,0292 | 773 | 343 |
| >1 secondary dx | 9873 | 1673 | 1983 |
|  |
| --- |
| Note: |
|  |
| 1 Controls |
| 2 Possible cases |
| 3 Probable cases |

**ICD-10 codes used to construct matrix above. Columns show count of individuals who have received at least one dx**

| ICD-10 sub-codes | Primary dx | Secondary dx |
| --- | --- | --- |
| **E10 Type 1 diabetes mellitus** | | |
| E10.0 With coma | 20 | 13 |
| E10.1 With ketoacidosis | 187 | 69 |
| E10.2 With renal complications | 10 | 71 |
| E10.3 With ophthalmic complications | 174 | 364 |
| E10.4 With neurological complications | 14 | 149 |
| E10.5 With peripheral circulatory complications | 56 | 57 |
| E10.6 With other specified complications | 16 | 27 |
| E10.7 With multiple complications | 0 | 8 |
| E10.8 With unspecified complications | 21 | 17 |
| E10.9 Without complications | 270 | 2,364 |

**Count of self-reported autoimmune condition**

| Self-reported disorder | Number of people |
| --- | --- |
| Type 1 diabetes | 364 |

**Count of self-reported prescription medications**

| Disease | Medication | Count |
| --- | --- | --- |
| Type 1 diabetes | insulin product | 3,925 |

## 1.4 Multiple Sclerosis

---

**Venn diagrams:** *Left:* probable cases as a subset of possible; *Right:* overlapping measures in the UK Biobank

**Matrix of ICD-10 primary and secondary dx - cells show number of individuals with each combination of dx**

|  | 0 primary dx | 1 primary dx | >1 primary dx |
| --- | --- | --- | --- |
| 0 secondary dx | 324,0741 | 1252 | 973 |
| 1 secondary dx | 2922 | 763 | 433 |
| >1 secondary dx | 3253 | 853 | 2093 |
|  |
| --- |
| Note: |
|  |
| 1 Controls |
| 2 Possible cases |
| 3 Probable cases |

**ICD-10 codes used to construct matrix above. Columns show count of individuals who have received at least one dx**

| ICD-10 sub-codes | Primary dx | Secondary dx |
| --- | --- | --- |
| **G35 Multiple sclerosis** | | |
| G35 Multiple sclerosis | 635 | 1,030 |

**Count of self-reported autoimmune condition**

| Self-reported disorder | Number of people |
| --- | --- |
| Multiple sclerosis | 1,426 |

**Count of self-reported prescription medications**

| Disease | Medication | Count |
| --- | --- | --- |
| Multiple Sclerosis | prednisolone | 2,310 |
|  | methotrexate | 2,096 |
|  | baclofen | 432 |
|  | mycophenolate | 174 |
|  | tacrolimus | 172 |
|  | prednisolone product | 139 |
|  | amantadine | 116 |
|  | ciclosporin | 50 |
|  | mtx - methotrexate | 36 |
|  | methylprednisolone | 19 |
|  | azt - azathioprine | 13 |
|  | myfortic 180mg gastro-resistant tablet | 13 |
|  | interferon beta-1a | 11 |
|  | glatiramer | 3 |
|  | interferon beta-1b | 3 |
|  | ciclosporin product | 1 |
|  | peginterferon alfa-2b | 1 |
|  | interferon beta-1b product | 0 |

## 1.5 Myasthenia Gravis

---

**Venn diagrams:** *Left:* probable cases as a subset of possible; *Right:* overlapping measures in the UK Biobank

**Matrix of ICD-10 primary and secondary dx - cells show number of individuals with each combination of dx**

|  | 0 primary dx | 1 primary dx | >1 primary dx |
| --- | --- | --- | --- |
| 0 secondary dx | 324,0741 | 352 | 73 |
| 1 secondary dx | 322 | 163 | 113 |
| >1 secondary dx | 443 | 143 | 223 |
|  |
| --- |
| Note: |
|  |
| 1 Controls |
| 2 Possible cases |
| 3 Probable cases |

**ICD-10 codes used to construct matrix above. Columns show count of individuals who have received at least one dx**

| ICD-10 sub-codes | Primary dx | Secondary dx |
| --- | --- | --- |
| **G70 Myasthenia gravis and other myoneural disorders** | | |
| G70.0 Myasthenia gravis | 105 | 139 |

**Count of self-reported autoimmune condition**

| Self-reported disorder | Number of people |
| --- | --- |
| Myasthenia gravis | 156 |

**Count of self-reported prescription medications**

| Disease | Medication | Count |
| --- | --- | --- |
| Myasthenia Gravis | prednisolone | 2,310 |
|  | methotrexate | 2,096 |
|  | mycophenolate | 174 |
|  | prednisolone product | 139 |
|  | ciclosporin | 50 |
|  | mtx - methotrexate | 36 |
|  | methylprednisolone | 19 |
|  | azt - azathioprine | 13 |
|  | myfortic 180mg gastro-resistant tablet | 13 |
|  | ciclosporin product | 1 |

## 1.6 Coeliac

---

**Venn diagrams:** *Left:* probable cases as a subset of possible; *Right:* overlapping measures in the UK Biobank

**Matrix of ICD-10 primary and secondary dx - cells show number of individuals with each combination of dx**

|  | 0 primary dx | 1 primary dx | >1 primary dx |
| --- | --- | --- | --- |
| 0 secondary dx | 324,0741 | 4002 | 693 |
| 1 secondary dx | 5002 | 1483 | 393 |
| >1 secondary dx | 3513 | 1343 | 553 |
|  |
| --- |
| Note: |
|  |
| 1 Controls |
| 2 Possible cases |
| 3 Probable cases |

**ICD-10 codes used to construct matrix above. Columns show count of individuals who have received at least one dx**

| ICD-10 sub-codes | Primary dx | Secondary dx |
| --- | --- | --- |
| **K90 Intestinal malabsorption** | | |
| K90.0 Coeliac disease | 845 | 1,227 |

**Count of self-reported autoimmune condition**

| Self-reported disorder | Number of people |
| --- | --- |
| Malabsorption/coeliac disease | 1,704 |

## 1.7 Inflammatory Bowel Disease

---

**Venn diagrams:** *Left:* probable cases as a subset of possible; *Right:* overlapping measures in the UK Biobank

**Matrix of ICD-10 primary and secondary dx - cells show number of individuals with each combination of dx**

|  | 0 primary dx | 1 primary dx | >1 primary dx |
| --- | --- | --- | --- |
| 0 secondary dx | 324,0741 | 9882 | 5173 |
| 1 secondary dx | 5972 | 3183 | 3823 |
| >1 secondary dx | 3283 | 2833 | 8133 |
|  |
| --- |
| Note: |
|  |
| 1 Controls |
| 2 Possible cases |
| 3 Probable cases |

**ICD-10 codes used to construct matrix above. Columns show count of individuals who have received at least one dx**

| ICD-10 sub-codes | Primary dx | Secondary dx |
| --- | --- | --- |
| **K50 Crohn disease [regional enteritis]** | | |
| K50.0 Crohn’s disease of small intestine | 264 | 166 |
| K50.1 Crohn’s disease of large intestine | 356 | 165 |
| K50.8 Other Crohn’s disease | 111 | 58 |
| K50.9 Crohn’s disease, unspecified | 814 | 1,013 |
| **K51 Ulcerative colitis** | | |
| K51.0 Ulcerative (chronic) enterocolitis | 101 | 34 |
| K51.1 Ulcerative (chronic) ileocolitis | 20 | 7 |
| K51.2 Ulcerative (chronic) proctitis | 328 | 81 |
| K51.3 Ulcerative (chronic) rectosigmoiditis | 174 | 34 |
| K51.4 Pseudopolyposis of colon | 99 | 99 |
| K51.5 Mucosal proctocolitis | 69 | 13 |
| K51.8 Other ulcerative colitis | 172 | 62 |
| K51.9 Ulcerative colitis, unspecified | 2,027 | 1,573 |

**Count of self-reported autoimmune condition**

| Self-reported disorder | Number of people |
| --- | --- |
| Crohns disease | 1,186 |
| Ulcerative colitis | 2,114 |

**Count of self-reported prescription medications**

| Disease | Medication | Count |
| --- | --- | --- |
| Inflammatory Bowel Disease | prednisolone | 2,310 |
|  | methotrexate | 2,096 |
|  | mesalazine | 570 |
|  | asacol 400mg e/c tablet | 563 |
|  | pentasa sr 250mg m/r tablet | 263 |
|  | asacol mr 400mg e/c tablet | 155 |
|  | prednisolone product | 139 |
|  | balsalazide disodium | 118 |
|  | humira 40mg injection solution 0.8ml prefilled syringe | 104 |
|  | olsalazine | 47 |
|  | adalimumab | 43 |
|  | mtx - methotrexate | 36 |
|  | methylprednisolone | 19 |
|  | azt - azathioprine | 13 |
|  | 5asa - mesalazine | 7 |

## 1.8 Psoriasis

---

**Venn diagrams:** *Left:* probable cases as a subset of possible; *Right:* overlapping measures in the UK Biobank

**Matrix of ICD-10 primary and secondary dx - cells show number of individuals with each combination of dx**

|  | 0 primary dx | 1 primary dx | >1 primary dx |
| --- | --- | --- | --- |
| 0 secondary dx | 324,0741 | 902 | 923 |
| 1 secondary dx | 9322 | 313 | 183 |
| >1 secondary dx | 3143 | 143 | 393 |
|  |
| --- |
| Note: |
|  |
| 1 Controls |
| 2 Possible cases |
| 3 Probable cases |

**ICD-10 codes used to construct matrix above. Columns show count of individuals who have received at least one dx**

| ICD-10 sub-codes | Primary dx | Secondary dx |
| --- | --- | --- |
| **L40 Psoriasis** | | |
| L40.0 Psoriasis vulgaris | 86 | 51 |
| L40.1 Generalised pustular psoriasis | 9 | 7 |
| L40.3 Pustulosis palmaris et plantaris | 8 | 8 |
| L40.4 Guttate psoriasis | 5 | 8 |
| L40.8 Other psoriasis | 16 | 17 |
| L40.9 Psoriasis, unspecified | 213 | 1,295 |

**Count of self-reported autoimmune condition**

| Self-reported disorder | Number of people |
| --- | --- |
| Psoriasis | 4,664 |

**Count of self-reported prescription medications**

| Disease | Medication | Count |
| --- | --- | --- |
| Psoriasis | beclometasone | 3,582 |
|  | prednisolone | 2,310 |
|  | methotrexate | 2,096 |
|  | beclomethasone | 1,594 |
|  | vitamin d product | 1,463 |
|  | betnovate cream | 1,384 |
|  | mometasone | 816 |
|  | hydrocortisone | 731 |
|  | diprobase cream | 644 |
|  | dermovate cream | 642 |
|  | betamethasone | 634 |
|  | dovobet ointment | 476 |
|  | eumovate cream | 427 |
|  | aqueous cream bp | 330 |
|  | hydrocortisone product | 271 |
|  | e45 cream | 269 |
|  | elocon cream | 263 |
|  | daktacort cream | 263 |
|  | trimovate ointment | 257 |
|  | prednisone | 251 |
|  | epaderm ointment | 243 |
|  | calcipotriol | 239 |
|  | dovonex 50micrograms/g cream | 222 |
|  | doublebase gel | 209 |
|  | fucibet cream | 204 |
|  | diprosalic ointment | 187 |
|  | mycophenolate | 174 |
|  | prednisolone product | 139 |
|  | dermol 500 lotion | 122 |
|  | clobetasone | 120 |
|  | humira 40mg injection solution 0.8ml prefilled syringe | 104 |
|  | synalar 1:10 cream | 101 |
|  | capasal shampoo | 100 |
|  | dovonex 50micrograms/g ointment | 97 |
|  | polytar liquid | 96 |
|  | clobetasol | 95 |
|  | mometasone furoate 0.1% ointment | 93 |
|  | clotrimazole | 91 |
|  | cetraben emollient cream | 88 |
|  | dovonex scalp solution | 81 |
|  | cetraben cream | 80 |
|  | dermol cream | 75 |
|  | oilatum emollient bath additive | 74 |
|  | diprobase ointment | 72 |
|  | locoid 0.1% cream | 69 |
|  | protopic 0.03% ointment | 67 |
|  | timodine cream | 67 |
|  | exorex lotion | 66 |
|  | hydromol cream | 66 |
|  | ketoconazole 2% shampoo | 65 |
|  | fucidin cream | 65 |
|  | mometasone furoate 0.1% lotion | 63 |
|  | cortisone | 62 |
|  | cyclosporin | 61 |
|  | neoral 10mg capsule | 59 |
|  | emulsifying ointment bp | 57 |
|  | betamethasone+calcipotriol | 55 |
|  | betacap scalp application | 53 |
|  | ciclosporin | 50 |
|  | neotigason 10mg capsule | 47 |
|  | nizoral 20mg/ml shampoo | 47 |
|  | cocois ointment | 46 |
|  | oilatum bath formula liquid bath additive | 44 |
|  | adalimumab | 43 |
|  | acitretin | 42 |
|  | calcitriol | 42 |
|  | cortisone product | 41 |
|  | alphosyl cream | 39 |
|  | balneum bath oil | 39 |
|  | fusidic acid | 39 |
|  | dermol 200 shower emollient | 38 |
|  | unguentum m cream | 37 |
|  | mtx - methotrexate | 36 |
|  | alphosyl hc cream | 35 |
|  | ketoconazole | 33 |
|  | metosyn 0.05% cream | 33 |
|  | liquid paraffin product | 31 |
|  | canesten hc cream | 31 |
|  | hydrocortisone+miconazole | 31 |
|  | tioconazole | 30 |
|  | coal tar product | 29 |
|  | alphaderm cream | 29 |
|  | cocois scalp ointment | 28 |
|  | diprosone cream | 28 |
|  | hydrocortisone+clotrimazole | 28 |
|  | emollient product | 27 |
|  | alphosyl shampoo | 26 |
|  | lotriderm cream | 26 |
|  | hydrocortistab 1% cream | 25 |
|  | liquid paraffin | 23 |
|  | salicylic acid product | 23 |
|  | curatoderm 4micrograms/g ointment | 22 |
|  | eurax cream | 22 |
|  | balneum plus cream | 21 |
|  | fucidin h cream | 21 |
|  | silkis 3micrograms/g ointment | 20 |
|  | calcitriol product | 20 |
|  | liquid paraffin+white soft paraffin 50%/50% ointment | 20 |
|  | balneum plus bath oil | 20 |
|  | nerisone 0.1% cream | 20 |
|  | polytar af liquid | 18 |
|  | clobetasone butyrate+neomycin sulphate | 18 |
|  | cutivate 0.05% cream | 17 |
|  | unguentum merck cream | 17 |
|  | nizoral 2% cream | 16 |
|  | hydrocortisyl 1% cream | 16 |
|  | cya - cyclosporin | 16 |
|  | elidel 1% cream | 16 |
|  | alphosyl 2 in 1 shampoo | 15 |
|  | clobetasol propionate+neomycin sulphate+nystatin | 15 |
|  | hydromol emollient bath additive | 15 |
|  | fucidin ointment | 14 |
|  | e45 lotion | 13 |
|  | tacrolimus monohydrate 0.03% ointment | 13 |
|  | polytar plus liquid | 12 |
|  | polytar emollient bath additive | 12 |
|  | aveeno lotion | 12 |
|  | dithrocream 0.1% cream | 11 |
|  | urea 10% cream | 11 |
|  | hc - hydrocortisone | 11 |
|  | eucerin 10% cream | 11 |
|  | tacalcitol | 10 |
|  | paraffin-white soft | 10 |
|  | haelan 0.0125% cream | 10 |
|  | salicylic acid | 9 |
|  | paraffin liquid | 9 |
|  | diprobath bath additive | 9 |
|  | e45 emollient bath oil | 9 |
|  | betamethasone+salicylic acid | 8 |
|  | emulsiderm emollient emulsion | 7 |
|  | selsun shampoo | 7 |
|  | calmurid hc cream | 7 |
|  | hydrocortisone+fusidic acid | 7 |
|  | eurax hc cream | 7 |
|  | dithranol | 6 |
|  | coal tar 4.3% shampoo | 6 |
|  | sebco ointment | 6 |
|  | psoriderm cream | 6 |
|  | bettamousse 0.12% foam | 6 |
|  | neutrogena dermatological cream | 5 |
|  | modrasone 0.05% cream | 5 |
|  | ung emuls - ungentum emulsificans | 5 |
|  | dermol cream 500g | 5 |
|  | mycophenolic acid 180mg gastro-resistant tablet | 5 |
|  | coal tar+salicylic acid ointment bp | 4 |
|  | t/gel shampoo | 4 |
|  | alphosyl lotion | 4 |
|  | dermacare cream 100ml | 4 |
|  | dithranol 0.2% ointment bp | 3 |
|  | dithrocream 2% cream | 3 |
|  | tazarotene | 3 |
|  | dithrocream forte 0.5% cream | 3 |
|  | dithrocream hp 1% cream | 3 |
|  | coal tar extract+hydrocortisone 3%/0.25% cream | 3 |
|  | coal tar extract 2% shampoo | 3 |
|  | efalizumab | 3 |
|  | clobetasone butyrate+oxytetracycline+nystatin | 3 |
|  | dithrocream 0.25% cream | 2 |
|  | psoriderm shampoo | 2 |
|  | hydrous ointment bp | 2 |
|  | dithranol+salicylic acid 0.25%/1.6% scalp gel | 2 |
|  | urea 10% lotion | 2 |
|  | oilatum bath formula liquid bath additive 150ml | 2 |
|  | zorac 0.05% aqueous gel | 1 |
|  | coal tar 40% bath emulsion | 1 |
|  | coal tar extract+allantoin 5%/2% cream | 1 |
|  | psoriderm bath emulsion | 1 |
|  | coal tar+lecithin 6/0.4% cream | 1 |
|  | strong coal tar solution+pine tar 5/5% gel | 1 |
|  | coal tar extract+allantoin 5%/2% lotion | 1 |
|  | psorin scalp gel | 1 |
|  | urea+lauromacrogols 5%/3% cream | 1 |
|  | dermacare cream 150ml | 1 |
|  | cortacream 1% band | 0 |
|  | methyl salicylate ointment bp | 0 |
|  | meted shampoo | 0 |

## 1.9 Ankylosing Spondylitis

---

**Venn diagrams:** *Left:* probable cases as a subset of possible; *Right:* overlapping measures in the UK Biobank

**Matrix of ICD-10 primary and secondary dx - cells show number of individuals with each combination of dx**

|  | 0 primary dx | 1 primary dx | >1 primary dx |
| --- | --- | --- | --- |
| 0 secondary dx | 324,0741 | 362 | 253 |
| 1 secondary dx | 2662 | 133 | 123 |
| >1 secondary dx | 1473 | 273 | 403 |
|  |
| --- |
| Note: |
|  |
| 1 Controls |
| 2 Possible cases |
| 3 Probable cases |

**ICD-10 codes used to construct matrix above. Columns show count of individuals who have received at least one dx**

| ICD-10 sub-codes | Primary dx | Secondary dx |
| --- | --- | --- |
| **M45 Ankylosing spondylitis** | | |
| M45 Ankylosing spondylitis | 101 | 303 |
| M45.X0 Ankylosing spondylitis (Multiple sites in spine) | 40 | 35 |
| M45.X1 Ankylosing spondylitis (Occipito-atlanto-axial region) | 0 | 4 |
| M45.X2 Ankylosing spondylitis (Cervical region) | 9 | 29 |
| M45.X4 Ankylosing spondylitis (Thoracic region) | 0 | 1 |
| M45.X6 Ankylosing spondylitis (Lumbar region) | 7 | 6 |
| M45.X7 Ankylosing spondylitis (Lumbosacral region) | 3 | 4 |
| M45.X8 Ankylosing spondylitis (Sacral and sacrococcygeal region) | 0 | 2 |
| M45.X9 Ankylosing spondylitis (Site unspecified) | 50 | 250 |

**Count of self-reported autoimmune condition**

| Self-reported disorder | Number of people |
| --- | --- |
| Ankylosing spondylitis | 1,118 |

**Count of self-reported prescription medications**

| Disease | Medication | Count |
| --- | --- | --- |
| Ankylosing Spondylitis | azathioprine | 841 |
|  | humira 40mg injection solution 0.8ml prefilled syringe | 104 |
|  | adalimumab | 43 |

## 1.10 Polymyalgia Rheumatica/Giant Cell Arteritis

---

**Venn diagrams:** *Left:* probable cases as a subset of possible; *Right:* overlapping measures in the UK Biobank

**Matrix of ICD-10 primary and secondary dx - cells show number of individuals with each combination of dx**

|  | 0 primary dx | 1 primary dx | >1 primary dx |
| --- | --- | --- | --- |
| 0 secondary dx | 324,0741 | 462 | 63 |
| 1 secondary dx | 5732 | 193 | 33 |
| >1 secondary dx | 3843 | 243 | 73 |
|  |
| --- |
| Note: |
|  |
| 1 Controls |
| 2 Possible cases |
| 3 Probable cases |

**ICD-10 codes used to construct matrix above. Columns show count of individuals who have received at least one dx**

| ICD-10 sub-codes | Primary dx | Secondary dx |
| --- | --- | --- |
| **M31 Other necrotizing vasculopathies** | | |
| M31.5 Giant cell arteritis with polymyalgia rheumatica | 11 | 33 |
| **M35 Other systemic involvement of connective tissue** | | |
| M35.3 Polymyalgia rheumatica | 96 | 1,002 |

**Count of self-reported autoimmune condition**

| Self-reported disorder | Number of people |
| --- | --- |
| Polymyalgia rheumatica | 871 |

**Count of self-reported prescription medications**

| Disease | Medication | Count |
| --- | --- | --- |
| Polymyalgia Rheumatica/Giant Cell Arteritis | prednisolone | 2,310 |
|  | methotrexate | 2,096 |
|  | prednisolone product | 139 |
|  | mtx - methotrexate | 36 |
|  | methylprednisolone | 19 |
|  | azt - azathioprine | 13 |

## 1.11 Psoriatic Arthritis

---

**Venn diagrams:** *Left:* probable cases as a subset of possible; *Right:* overlapping measures in the UK Biobank

**Matrix of ICD-10 primary and secondary dx - cells show number of individuals with each combination of dx**

|  | 0 primary dx | 1 primary dx | >1 primary dx |
| --- | --- | --- | --- |
| 0 secondary dx | 324,0741 | 112 | 83 |
| 1 secondary dx | 212 | 693 | 03 |
| >1 secondary dx | 3733 | 683 | 1163 |
|  |
| --- |
| Note: |
|  |
| 1 Controls |
| 2 Possible cases |
| 3 Probable cases |

**ICD-10 codes used to construct matrix above. Columns show count of individuals who have received at least one dx**

| ICD-10 sub-codes | Primary dx | Secondary dx |
| --- | --- | --- |
| **L40 Psoriasis** | | |
| L40.5 Arthropathic psoriasis | 258 | 554 |
| **M07 Psoriatic and enteropathic arthropathies** | | |
| M07.3 Other psoriatic arthropathies | 7 | 354 |
| M07.30 Other psoriatic arthropathies (Multiple sites) | 6 | 139 |
| M07.31 Other psoriatic arthropathies (Shoulder region) | 0 | 7 |
| M07.32 Other psoriatic arthropathies (Upper arm) | 0 | 1 |
| M07.33 Other psoriatic arthropathies (Forearm) | 0 | 5 |
| M07.34 Other psoriatic arthropathies (Hand) | 0 | 19 |
| M07.35 Other psoriatic arthropathies (Pelvic region and thigh) | 1 | 15 |
| M07.36 Other psoriatic arthropathies (Lower leg) | 5 | 29 |
| M07.37 Other psoriatic arthropathies (Ankle and foot) | 2 | 21 |
| M07.38 Other psoriatic arthropathies (Other) | 0 | 7 |
| M07.39 Other psoriatic arthropathies (Site unspecified) | 7 | 260 |

**Count of self-reported autoimmune condition**

| Self-reported disorder | Number of people |
| --- | --- |
| Psoriatic arthropathy | 766 |

**Count of self-reported prescription medications**

| Disease | Medication | Count |
| --- | --- | --- |
| Psoriatic Arthritis | prednisolone | 2,310 |
|  | methotrexate | 2,096 |
|  | azathioprine | 841 |
|  | leflunomide | 158 |
|  | prednisolone product | 139 |
|  | humira 40mg injection solution 0.8ml prefilled syringe | 104 |
|  | adalimumab | 43 |
|  | mtx - methotrexate | 36 |
|  | methylprednisolone | 19 |
|  | arava 10mg tablet | 9 |
|  | arava 20mg tablet | 6 |

## 1.12 Rheumatoid Arthritis

---

**Venn diagrams:** *Left:* probable cases as a subset of possible; *Right:* overlapping measures in the UK Biobank

**Matrix of ICD-10 primary and secondary dx - cells show number of individuals with each combination of dx**

|  | 0 primary dx | 1 primary dx | >1 primary dx |
| --- | --- | --- | --- |
| 0 secondary dx | 324,0741 | 3762 | 1843 |
| 1 secondary dx | 1,4252 | 1213 | 1663 |
| >1 secondary dx | 9773 | 2543 | 5763 |
|  |
| --- |
| Note: |
|  |
| 1 Controls |
| 2 Possible cases |
| 3 Probable cases |

**ICD-10 codes used to construct matrix above. Columns show count of individuals who have received at least one dx**

| ICD-10 sub-codes | Primary dx | Secondary dx |
| --- | --- | --- |
| **M05 Seropositive rheumatoid arthritis** | | |
| M05.0 Felty’s syndrome | 1 | 4 |
| M05.00 Felty’s syndrome (Multiple sites) | 2 | 4 |
| M05.09 Felty’s syndrome (Site unspecified) | 1 | 4 |
| M05.1 Rheumatoid lung disease | 9 | 14 |
| M05.10 Rheumatoid lung disease (Multiple sites) | 3 | 3 |
| M05.19 Rheumatoid lung disease (Site unspecified) | 3 | 7 |
| M05.2 Rheumatoid vasculitis | 8 | 6 |
| M05.20 Rheumatoid vasculitis (Multiple sites) | 5 | 3 |
| M05.26 Rheumatoid vasculitis (Lower leg) | 1 | 0 |
| M05.28 Rheumatoid vasculitis (Other) | 1 | 0 |
| M05.29 Rheumatoid vasculitis (Site unspecified) | 4 | 5 |
| M05.3 Rheumatoid arthritis with involvement of other organs and systems | 1 | 2 |
| M05.30 Rheumatoid arthritis with involvement of other organs and systems (Multiple sites) | 1 | 1 |
| M05.38 Rheumatoid arthritis with involvement of other organs and systems (Other) | 0 | 1 |
| M05.8 Other seropositive rheumatoid arthritis | 12 | 6 |
| M05.80 Other seropositive rheumatoid arthritis (Multiple sites) | 8 | 4 |
| M05.82 Other seropositive rheumatoid arthritis (Upper arm) | 1 | 0 |
| M05.83 Other seropositive rheumatoid arthritis (Forearm) | 3 | 0 |
| M05.84 Other seropositive rheumatoid arthritis (Hand) | 2 | 1 |
| M05.86 Other seropositive rheumatoid arthritis (Lower leg) | 1 | 0 |
| M05.87 Other seropositive rheumatoid arthritis (Ankle and foot) | 1 | 0 |
| M05.88 Other seropositive rheumatoid arthritis (Other) | 1 | 0 |
| M05.89 Other seropositive rheumatoid arthritis (Site unspecified) | 5 | 2 |
| M05.9 Seropositive rheumatoid arthritis, unspecified | 90 | 107 |
| M05.90 Seropositive rheumatoid arthritis, unspecified (Multiple sites) | 164 | 101 |
| M05.91 Seropositive rheumatoid arthritis, unspecified (Shoulder region) | 4 | 3 |
| M05.92 Seropositive rheumatoid arthritis, unspecified (Upper arm) | 8 | 2 |
| M05.93 Seropositive rheumatoid arthritis, unspecified (Forearm) | 7 | 3 |
| M05.94 Seropositive rheumatoid arthritis, unspecified (Hand) | 11 | 5 |
| M05.95 Seropositive rheumatoid arthritis, unspecified (Pelvic region and thigh) | 7 | 3 |
| M05.96 Seropositive rheumatoid arthritis, unspecified (Lower leg) | 17 | 3 |
| M05.97 Seropositive rheumatoid arthritis, unspecified (Ankle and foot) | 19 | 4 |
| M05.98 Seropositive rheumatoid arthritis, unspecified (Other) | 2 | 1 |
| M05.99 Seropositive rheumatoid arthritis, unspecified (Site unspecified) | 68 | 81 |
| **M06 Other rheumatoid arthritis** | | |
| M06.0 Seronegative rheumatoid arthritis | 57 | 115 |
| M06.00 Seronegative rheumatoid arthritis (Multiple sites) | 105 | 94 |
| M06.01 Seronegative rheumatoid arthritis (Shoulder region) | 7 | 1 |
| M06.02 Seronegative rheumatoid arthritis (Upper arm) | 4 | 1 |
| M06.03 Seronegative rheumatoid arthritis (Forearm) | 4 | 1 |
| M06.04 Seronegative rheumatoid arthritis (Hand) | 3 | 5 |
| M06.05 Seronegative rheumatoid arthritis (Pelvic region and thigh) | 4 | 2 |
| M06.06 Seronegative rheumatoid arthritis (Lower leg) | 16 | 6 |
| M06.07 Seronegative rheumatoid arthritis (Ankle and foot) | 10 | 2 |
| M06.08 Seronegative rheumatoid arthritis (Other) | 1 | 0 |
| M06.09 Seronegative rheumatoid arthritis (Site unspecified) | 33 | 104 |
| M06.1 Adult-onset Still’s disease | 6 | 4 |
| M06.10 Adult-onset Still’s disease (Multiple sites) | 2 | 3 |
| M06.16 Adult-onset Still’s disease (Lower leg) | 1 | 0 |
| M06.19 Adult-onset Still’s disease (Site unspecified) | 3 | 3 |
| M06.21 Rheumatoid bursitis (Shoulder region) | 1 | 0 |
| M06.22 Rheumatoid bursitis (Upper arm) | 1 | 0 |
| M06.27 Rheumatoid bursitis (Ankle and foot) | 1 | 0 |
| M06.3 Rheumatoid nodule | 19 | 3 |
| M06.30 Rheumatoid nodule (Multiple sites) | 8 | 4 |
| M06.32 Rheumatoid nodule (Upper arm) | 20 | 5 |
| M06.33 Rheumatoid nodule (Forearm) | 5 | 3 |
| M06.34 Rheumatoid nodule (Hand) | 48 | 13 |
| M06.36 Rheumatoid nodule (Lower leg) | 2 | 2 |
| M06.37 Rheumatoid nodule (Ankle and foot) | 22 | 10 |
| M06.39 Rheumatoid nodule (Site unspecified) | 2 | 0 |
| M06.4 Inflammatory polyarthropathy | 23 | 19 |
| M06.40 Inflammatory polyarthropathy (Multiple sites) | 32 | 19 |
| M06.41 Inflammatory polyarthropathy (Shoulder region) | 2 | 1 |
| M06.43 Inflammatory polyarthropathy (Forearm) | 2 | 0 |
| M06.44 Inflammatory polyarthropathy (Hand) | 2 | 0 |
| M06.45 Inflammatory polyarthropathy (Pelvic region and thigh) | 1 | 1 |
| M06.46 Inflammatory polyarthropathy (Lower leg) | 9 | 2 |
| M06.47 Inflammatory polyarthropathy (Ankle and foot) | 2 | 0 |
| M06.49 Inflammatory polyarthropathy (Site unspecified) | 40 | 19 |
| M06.8 Other specified rheumatoid arthritis | 10 | 3 |
| M06.80 Other specified rheumatoid arthritis (Multiple sites) | 3 | 6 |
| M06.81 Other specified rheumatoid arthritis (Shoulder region) | 2 | 0 |
| M06.82 Other specified rheumatoid arthritis (Upper arm) | 1 | 1 |
| M06.84 Other specified rheumatoid arthritis (Hand) | 0 | 1 |
| M06.85 Other specified rheumatoid arthritis (Pelvic region and thigh) | 1 | 0 |
| M06.86 Other specified rheumatoid arthritis (Lower leg) | 1 | 0 |
| M06.87 Other specified rheumatoid arthritis (Ankle and foot) | 2 | 0 |
| M06.88 Other specified rheumatoid arthritis (Other) | 5 | 0 |
| M06.89 Other specified rheumatoid arthritis (Site unspecified) | 6 | 4 |
| M06.9 Rheumatoid arthritis, unspecified | 513 | 1,682 |
| M06.90 Rheumatoid arthritis, unspecified (Multiple sites) | 449 | 697 |
| M06.91 Rheumatoid arthritis, unspecified (Shoulder region) | 77 | 29 |
| M06.92 Rheumatoid arthritis, unspecified (Upper arm) | 42 | 4 |
| M06.93 Rheumatoid arthritis, unspecified (Forearm) | 41 | 25 |
| M06.94 Rheumatoid arthritis, unspecified (Hand) | 116 | 89 |
| M06.95 Rheumatoid arthritis, unspecified (Pelvic region and thigh) | 55 | 15 |
| M06.96 Rheumatoid arthritis, unspecified (Lower leg) | 137 | 65 |
| M06.97 Rheumatoid arthritis, unspecified (Ankle and foot) | 114 | 54 |
| M06.98 Rheumatoid arthritis, unspecified (Other) | 13 | 19 |
| M06.99 Rheumatoid arthritis, unspecified (Site unspecified) | 475 | 1,868 |

**Count of self-reported autoimmune condition**

| Self-reported disorder | Number of people |
| --- | --- |
| Rheumatoid arthritis | 4,364 |

**Count of self-reported prescription medications**

| Disease | Medication | Count |
| --- | --- | --- |
| Rheumatoid Arthritis | prednisolone | 2,310 |
|  | methotrexate | 2,096 |
|  | azathioprine | 841 |
|  | hydroxychloroquine | 590 |
|  | leflunomide | 158 |
|  | prednisolone product | 139 |
|  | humira 40mg injection solution 0.8ml prefilled syringe | 104 |
|  | adalimumab | 43 |
|  | mtx - methotrexate | 36 |
|  | methylprednisolone | 19 |
|  | azt - azathioprine | 13 |
|  | arava 10mg tablet | 9 |
|  | arava 20mg tablet | 6 |
|  | gold product | 6 |
|  | sodium aurothiomalate | 4 |
|  | gold sodium thiomalate | 2 |

## 1.13 Sjögren Syndrome

---

**Venn diagrams:** *Left:* probable cases as a subset of possible; *Right:* overlapping measures in the UK Biobank

**Matrix of ICD-10 primary and secondary dx - cells show number of individuals with each combination of dx**

|  | 0 primary dx | 1 primary dx | >1 primary dx |
| --- | --- | --- | --- |
| 0 secondary dx | 324,0741 | 222 | 43 |
| 1 secondary dx | 1862 | 113 | 83 |
| >1 secondary dx | 2043 | 193 | 153 |
|  |
| --- |
| Note: |
|  |
| 1 Controls |
| 2 Possible cases |
| 3 Probable cases |

**ICD-10 codes used to construct matrix above. Columns show count of individuals who have received at least one dx**

| ICD-10 sub-codes | Primary dx | Secondary dx |
| --- | --- | --- |
| **M35 Other systemic involvement of connective tissue** | | |
| M35.0 Sicca syndrome [Sjogren] | 79 | 443 |

**Count of self-reported autoimmune condition**

| Self-reported disorder | Number of people |
| --- | --- |
| Sjogren’s syndrome/sicca syndrome | 373 |

**Count of self-reported prescription medications**

| Disease | Medication | Count |
| --- | --- | --- |
| Sjögren Syndrome | prednisolone | 2,310 |
|  | methotrexate | 2,096 |
|  | hydroxychloroquine | 590 |
|  | prednisolone product | 139 |
|  | mtx - methotrexate | 36 |
|  | methylprednisolone | 19 |
|  | azt - azathioprine | 13 |

## 1.14 Systemic Lupus Erythematosus

---

**Venn diagrams:** *Left:* probable cases as a subset of possible; *Right:* overlapping measures in the UK Biobank

**Matrix of ICD-10 primary and secondary dx - cells show number of individuals with each combination of dx**

|  | 0 primary dx | 1 primary dx | >1 primary dx |
| --- | --- | --- | --- |
| 0 secondary dx | 324,0741 | 432 | 113 |
| 1 secondary dx | 1442 | 83 | 63 |
| >1 secondary dx | 1283 | 273 | 343 |
|  |
| --- |
| Note: |
|  |
| 1 Controls |
| 2 Possible cases |
| 3 Probable cases |

**ICD-10 codes used to construct matrix above. Columns show count of individuals who have received at least one dx**

| ICD-10 sub-codes | Primary dx | Secondary dx |
| --- | --- | --- |
| **L93 Lupus erythematosus** | | |
| L93.0 Discoid lupus erythematosus | 20 | 91 |
| L93.1 Subacute cutaneous lupus erythematosus | 2 | 6 |
| L93.2 Other local lupus erythematosus | 3 | 8 |
| **M32 Systemic lupus erythematosus** | | |
| M32.1 Systemic lupus erythematosus with organ or system involvement | 30 | 32 |
| M32.8 Other forms of systemic lupus erythematosus | 5 | 6 |
| M32.9 Systemic lupus erythematosus, unspecified | 83 | 280 |
| M32.90 Systemic lupus erythematosus, unspecified, Multiple sites | 1 | 3 |

**Count of self-reported autoimmune condition**

| Self-reported disorder | Number of people |
| --- | --- |
| Systemic lupus erythematosis/sle | 453 |

**Count of self-reported prescription medications**

| Disease | Medication | Count |
| --- | --- | --- |
| Systemic Lupus Erythematosus | prednisolone | 2,310 |
|  | methotrexate | 2,096 |
|  | hydroxychloroquine | 590 |
|  | mycophenolate | 174 |
|  | tacrolimus | 172 |
|  | prednisolone product | 139 |
|  | ciclosporin | 50 |
|  | mtx - methotrexate | 36 |
|  | methylprednisolone | 19 |
|  | azt - azathioprine | 13 |
|  | myfortic 180mg gastro-resistant tablet | 13 |
|  | tacrolimus monohydrate 0.03% ointment | 13 |
|  | ciclosporin product | 1 |
